# TRPM4 is overexpressed in breast cancer associated with estrogen response and epithelial-mesenchymal transition gene sets

**DOI:** 10.1371/journal.pone.0233884

**Published:** 2020-06-02

**Authors:** Kah Keng Wong, Faezahtul Arbaeyah Hussain

**Affiliations:** 1 Department of Immunology, School of Medical Sciences, Universiti Sains Malaysia, Kubang Kerian, Kelantan, Malaysia; 2 Department of Pathology, School of Medical Sciences, Universiti Sains Malaysia, Kubang Kerian, Kelantan, Malaysia; Qatar University College of Medicine, QATAR

## Abstract

Ion channels form an important class of drug targets in malignancies. Transient receptor potential cation channel subfamily M member 4 (TRPM4) plays oncological roles in various solid tumors. Herein, we examined TRPM4 protein expression profile by immunohistochemistry (IHC) in breast cancer cases compared with normal breast ducts, its association with clinico-demographical parameters, and its potential function in breast cancers by Gene Set Enrichment Analysis (GSEA). Data-mining demonstrated that *TRPM4* transcript levels were significantly higher in The Cancer Genome Atlas series of breast cancer cases (n = 1,085) compared with normal breast tissues (n = 112) (*p* = 1.03 x 10^−11^). Our IHC findings in tissue microarrays showed that TRPM4 protein was overexpressed in breast cancers (n = 83/99 TRPM4^+^; 83.8%) compared with normal breast ducts (n = 5/10 TRPM4^+^; 50%) (*p* = 0.022). Higher TRPM4 expression (median frequency cut-off) was significantly associated with higher lymph node status (N1-N2 vs N0; *p* = 0.024) and higher stage (IIb-IIIb vs I-IIa; *p* = 0.005). GSEA evaluation in three independent gene expression profiling (GEP) datasets of breast cancer cases (GSE54002, n = 417; GSE20685, n = 327; GSE23720, n = 197) demonstrated significant association of *TRPM4* transcript expression with estrogen response and epithelial-mesenchymal transition (EMT) gene sets (*p*<0.01 and false discovery rate<0.05). These gene sets were not enriched in GEP datasets of normal breast epithelium cases (GSE10797, n = 5; GSE9574, n = 15; GSE20437, n = 18). In conclusion, TRPM4 protein expression is upregulated in breast cancers associated with worse clinico-demographical parameters, and TRPM4 potentially regulates estrogen receptor signaling and EMT progression in breast cancer.

## Introduction

Ion channels form an important class of therapeutic target where they account for nearly one-fifth of all human druggable proteins [1]. Ion channels contribute to various malignant phenotypes of cancer cells through regulating the transport of the universal signaling ion calcium (Ca^2+^) [2]. Transient receptor potential (TRP) ion channels were identified in *Drosophila* in which mutated *trp* and *trpl* genes led to transient depolarization as well as receptor potential [3]. On the basis of sequence homology, mammalian TRP channels can be categorized into six subfamilies including the TRPM group of ion channels [4]. The TRPM subfamily consists of eight ion channel members (TRPM1-8) where each contains six transmembrane domains and a loop that forms the channel’s pore [5, 6].

Transient receptor potential melastatin 4 (TRPM4) is a non-selective cation channel activated by increased cytoplasmic Ca^2+^ to allow transport of monovalent cations such as Na^+^, K^+^, Cs^+^ and Li^+^ but impermeable to Ca^2+^ cation [7–9]. TRPM4 activation triggers cell depolarization that reduces the driving force for Ca^2+^ transport required to modulate various physiological processes including vasoconstriction of cerebral arteries, insulin secretion, and migration of immune cells [10–13]. In diseases, TRPM4 is frequently implicated in cardiovascular disorders [14] and recently implicated in malignancies [15, 16].

Independent investigations have shown the oncogenic roles of TRPM4 in prostate cancer. TRPM4 mRNA and protein levels were overexpressed in prostate cancer tissues compared with non-malignant pancreatic ducts [17, 18], and its overexpression conferred increased risk of biochemical recurrence in patients with prostate cancer [18]. TRPM4 expression induced the proliferation, migration and invasion of prostate cancer cells [17, 19–21] via TRPM4-mediated activation of β-catenin signaling pathway and epithelial-mesenchymal transition (EMT) [20, 21]. TRPM4 is also overexpressed in diffuse large B-cell lymphoma associated with worse survival [22], cervical cancer [23] and colorectal cancer where it could induce proliferation and invasion of colorectal cancer cells [24].

Breast cancer is the most common cancer among women globally where it accounts for approximately 25% of all female cancers [25, 26]. It is the leading cause of cancer death in women worldwide despite improvements in hormone and targeted therapies [26]. The members of TRPM ion channel family such as TRPM2, TRPM7 and TRPM8 play vital roles in the growth, survival and metastasis of breast cancer cells, while somatic mutations affecting *TRPM6* occur in breast cancer patients [15]. We thus set out to investigate the expression profile of TRPM4 in breast cancers, and to examine the potential roles of TRPM4 in the disease based on its expression profile in gene expression profiling (GEP) datasets of breast cancer tissues compared with normal breast epithelium tissues.

## Materials and methods

### Tissues and tissue microarrays (TMAs)

Two independent panels of formalin-fixed paraffin-embedded (FFPE) TMAs of breast cancer cases were obtained from US Biomax (Rockville, MD, USA). The first panel (catalogue no: BR1009) consisted of breast cancer (n = 40) and normal breast tissues adjacent to tumor (NBT; n = 7), while the second panel (catalogue no: BR1503f) consisted of breast cancer (n = 59), ductal carcinoma *in situ* (DCIS) *i*.*e*. pre-cancerous lesion of glandular tissues (n = 6), fibroadenoma *i*.*e*. benign proliferative lesion of both glandular and stroma components (n = 3), and NBT (n = 3). All TMAs contained duplicate cores per case and the following clinico-demographical and pathological data: Age, tumor size, lymph node status, tumor grade, ER, PR and HER2 protein status, and breast cancer subtypes. Furthermore, the first and second TMA panel contained tumor stage and Ki-67 frequency data, respectively. In addition to the TMAs, whole tissue sections of appendix from an adult female patient with appendicitis were obtained from Department of Pathology, Universiti Sains Malaysia, FFPE blocks archive and used as positive controls for IHC staining. All other data pertaining to the appendicitis patient who sought treatment in November 2018 were fully anonymized before the tissue sections were retrieved in July 2019. The study procedures were approved by the Human Research Ethics Committee of Universiti Sains Malaysia (JEPeM) (approved ethics code: USM/JEPeM/18050232). All procedures carried out in this study involving human tissue samples were in accordance with the 1964 Declaration of Helsinki and its later updates, and with the institutional ethical standards. All experimental protocols were conducted according to the institutional relevant guidelines and regulations. All tissue samples in the TMAs obtained from US Biomax were anonymous while the appendix tissue samples were linked-anonymized archival FFPE specimens, and individual consent was not required for this study.

### Immunohistochemistry (IHC)

Tissue sections were incubated at 60°C for 10 min to facilitate tissue adherence onto the slides before deparaffinization in two changes of xylene substitute (Sigma-Aldrich Co., St Louis, MO, USA) each for 15 min. This was followed by serial rehydration in graded ethanol (GmbH, Hamburg, Germany) from 100% ethanol followed by 70%, 50% and 30% ethanol, and finally in distilled water. Heat-mediated antigen retrieval was conducted in Tris-EDTA buffer (pH 9.0) using a microwave pressure cooker for 10 min followed by incubation with a mouse anti-TRPM4 monoclonal antibody (clone 10H5; Abcam, Cambridge, UK) at 1:500 dilution (1.536 μg/ml) for one hour at room temperature. Binding of the anti-TRPM4 antibody was detected using HRP-conjugated secondary anti-mouse/rabbit antibody from EnVision^TM^ detection system (DakoCytomation, Carpinteria, CA, USA) for 30 min and developed with DAB as the chromogen for 5 min. The sections were counterstained with fresh Gill No. 2 hematoxylin solution (Sigma Aldrich) for 10 sec and mounted with the VectaMount^TM^ (Vector Labs, Burlingame, California) non-aqueous mounting medium.

### Pathologic interpretation

Interpretation of the IHC staining was conducted by an experienced histopathologist F.A.H and researcher K.K.W independently blinded to the clinico-demographical and pathological data. The frequency of TRPM4 staining in tumor cells was scored in 10% increments and cases with ≥20% discrepancy were resolved under a joint microscope. Intensity of TRPM4 staining was scored as negative, weak, moderate, or strong.

### Gene Expression Profiling (GEP) datasets and Gene Set Enrichment Analysis (GSEA)

*TRPM4* transcript expression values (z-scores) from the The Cancer Genome Atlas (TCGA) dataset of breast cancer cases (n = 500) [27] matched for gender (females) and age range (27–81 years old) with the TMA series were obtained from the cBioPortal database (https://www.cbioportal.org/) [28, 29]. The clinico-demographical and pathological parameters retrieved from the dataset consisted of age, lymph node status, stage, ER, PR and HER2 status, and breast cancer subtypes. Microarray GEP datasets of normal breast epithelium from reduction mammoplasty individuals were obtained from GSE10797 (n = 5) [30], GSE9574 (n = 15) [31] and GSE20437 (n = 18) [32], while microarray GEP datasets of breast cancer patients were obtained from GSE54002 (n = 417) [33], GSE20685 (n = 327) [34] and GSE23720 (n = 197) [35] available on the Gene Expression Omnibus database (http://www.ncbi.nlm.nih.gov/geo/). All microarray GEP datasets were processed using the geWorkbench platform as described previously [36–38] where each GEP dataset’s values were log_2_-transformed, z-scores obtained (mean and variance normalization), and the z-scores were scaled to be within -3 (minimum) and 3 (maximum). Gene Set Enrichment Analysis (GSEA) [39] according to *TRPM4* expression (Affymetrix probe ID: 219360_s_at) and the Hallmark collection of the Molecular Signatures Database (MSigDB) [40] was conducted in each of the GEP dataset independently by using Pearson correlation as the gene-ranking metric and permutated using the gene sets permutation function [41].

### Statistical analysis

The distributions of clinico-demographical and pathological variables of the breast cancer patients were compared in terms of TRPM4 frequency or intensity of IHC staining using the χ^2^-test or Fisher’s exact test (used when more than 20% of cells have expected frequencies of below five) [42] (SPSS Statistics v22; IBM, Armonk, NY, USA). For TRPM4 frequency, the median threshold (*i*.*e*. 50%) was used as the cut-off, and the group of patients with ≥50% TRPM4 frequency was compared with patients harboring <50% TRPM4 frequency. For TRPM4 intensity, patients with negative or weak TRPM4 intensity were grouped together to be compared with patients harboring moderate or strong TRPM4 intensity. All *p*-values were two-tailed and values <0.05 were considered statistically significant. Finally, *a priori* sample size and power calculation was not performed due to TRPM4 protein expression profile in breast cancer cases and the associations examined had not been reported before. Hence, we were unable to estimate the effect of sample sizes according to other data.

## Results

### TRPM4 expression profile in breast cancer

**Fig 1:**
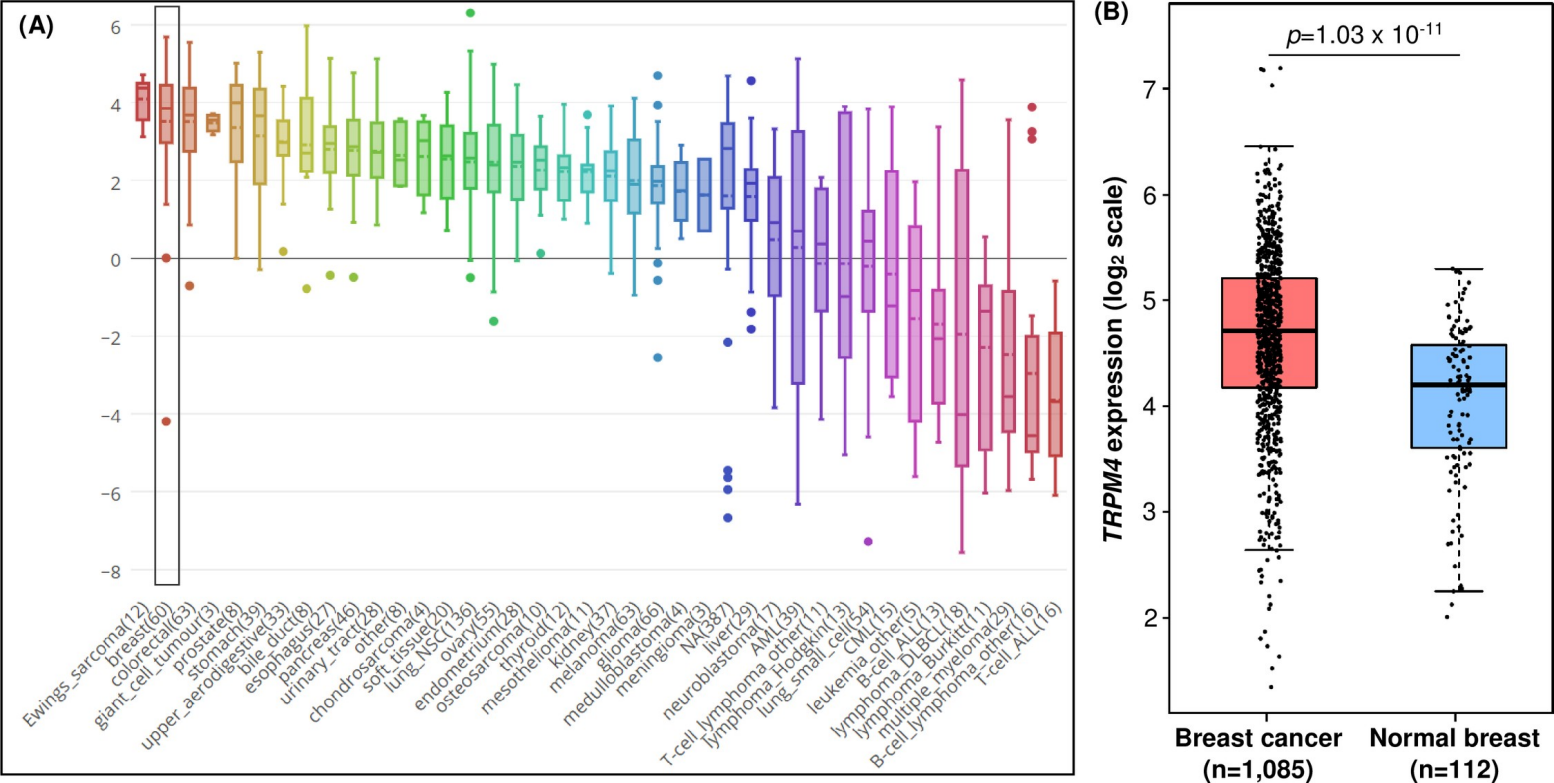
*TRPM4* transcript expression profile in cancer cell lines and breast cancer cases. (A) *TRPM4* transcript levels in cancer cell lines and each cancer type is represented by multiple cell lines (numbers in bracket denote total number of cell lines). Inset bracket denotes *TRPM4* levels in breast cancer cell lines (n = 60). Data derived from the CCLE database (https://portals.broadinstitute.org/ccle). (B) *TRPM4* transcript levels in TCGA series of breast cancer cases (n = 1,085) compared with normal breast tissues (n = 112) according to GEPIA2 (http://gepia2.cancer-pku.cn/) database.

Our initial data-mining showed that *TRPM4* transcript was frequently expressed in breast cancer cell lines according to the Cancer Cell Line Encyclopedia (CCLE) database (https://portals.broadinstitute.org/ccle) [43]. *TRPM4* transcript was more highly expressed in breast cancer cell lines (n = 60) compared with 38 other cancer types with the exception of Ewing’s sarcoma (n = 12) with higher *TRPM4* expression (Fig 1A). In The Cancer Genome Atlas (TCGA) breast cancer cases as curated by Gene Expression Profiling Interactive Analysis 2 database (http://gepia2.cancer-pku.cn/) [44], *TRPM4* transcript levels were significantly higher in breast cancer cases (n = 1,085) compared with normal breast tissues (n = 112) (p = 1.03 x 10^−11^) (Fig 1B).

**Fig 2:**
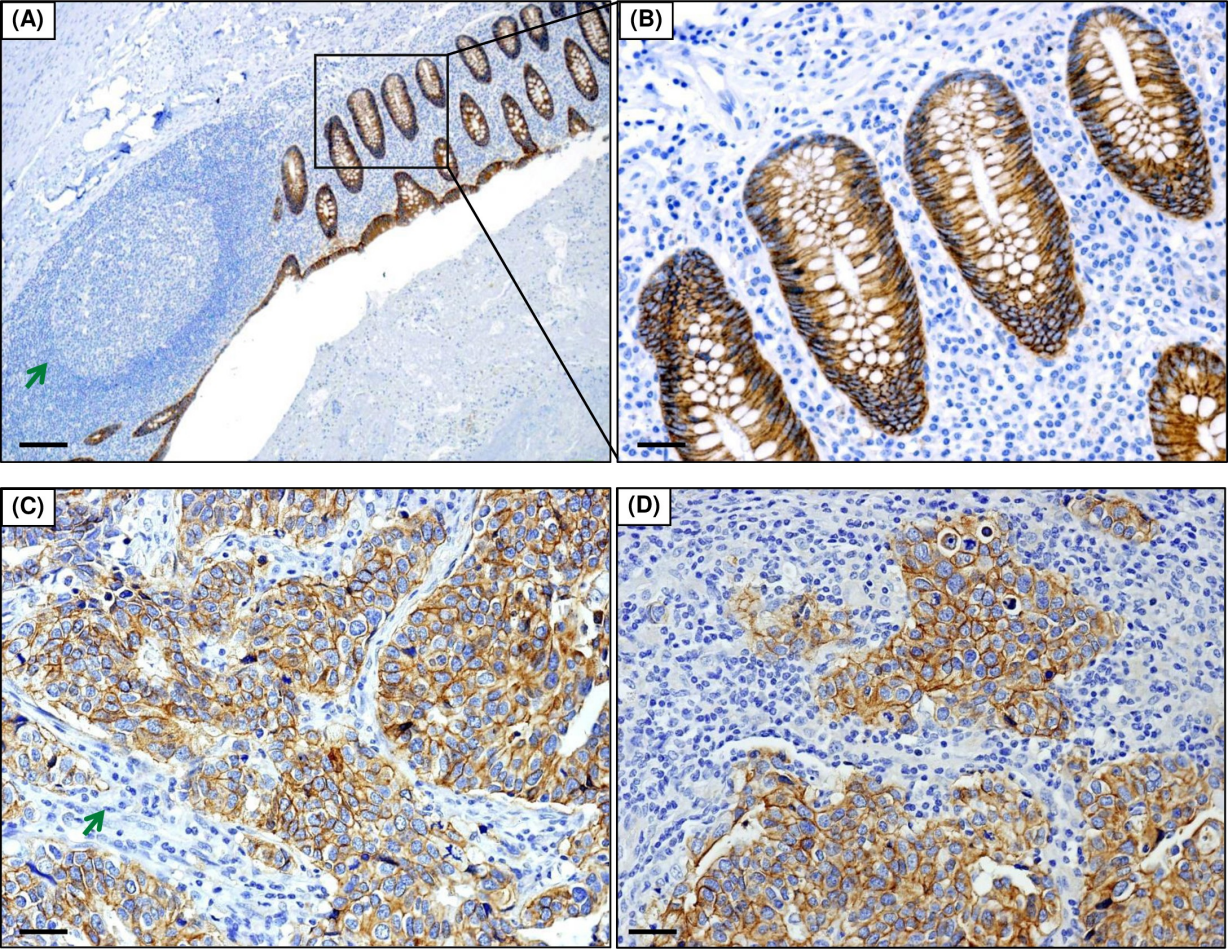
TRPM4 protein expression in representative control tissues and breast cancer cases. (A) TRPM4 expression by IHC in appendix as the control tissues (magnification: 40x). The green arrow shows the germinal centre negative for TRPM4 expression. (B) Higher magnification (200x) of the inset in (A). (C-D) Representative breast cancer tissues with high TRPM4 expression in breast cancer cells while negative in surrounding lymphocytes or stromal cells (green arrows). Scale bar for (A): 200 μm; Scale bar for (B-D): 50 μm.

IHC of the TMAs yielded 99 assessable breast cancer cases after exclusion of cases without sufficient breast cancer cells or missing cores. The IHC yielded strong cytoplasmic and membranous staining of TRPM4 in glandular cells of the appendix as control tissues (Fig 2A and 2B). The surrounding lymphocytes and adjacent lymphoid follicle with a germinal centre in the appendix were negative for TRPM4, consistent with previous findings [22]. All breast cancers yielded cytoplasmic and membranous staining of TRPM4 specifically in breast cancer cells, and negative in surrounding lymphocytes or stromal cells (representative pictures in Fig 2C and 2D).

**Fig 3:**
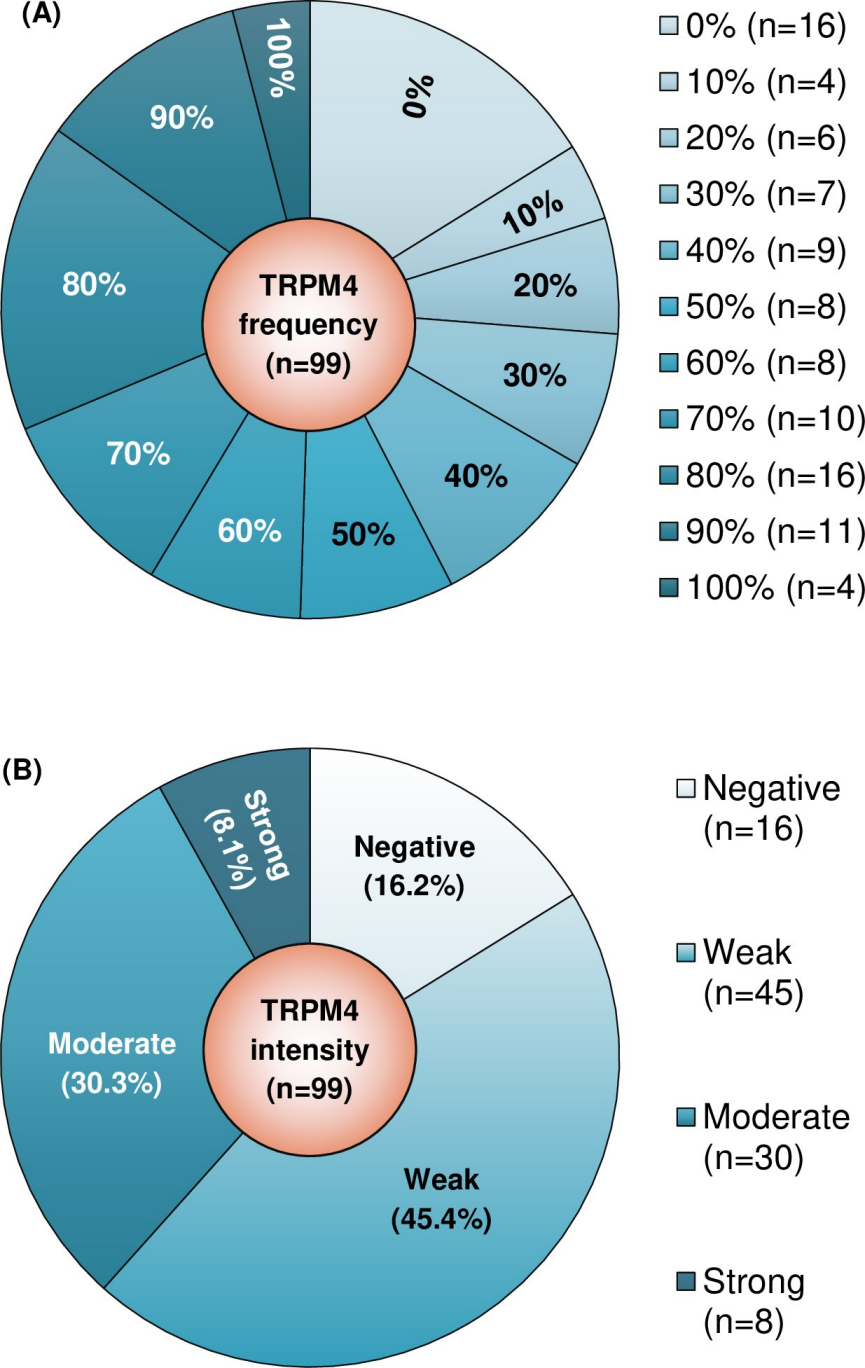
TRPM4 protein expression profile in breast cancer cases in terms of TRPM4 frequency and intensity. (A) Distribution of breast cancer cases (n = 99) according to TRPM4 frequency at every 10% increment. (B) Distribution of breast cancer cases (n = 99) according to TRPM4 intensity (weak, moderate or strong) and the proportion of cases for each intensity.

**Fig 4:**
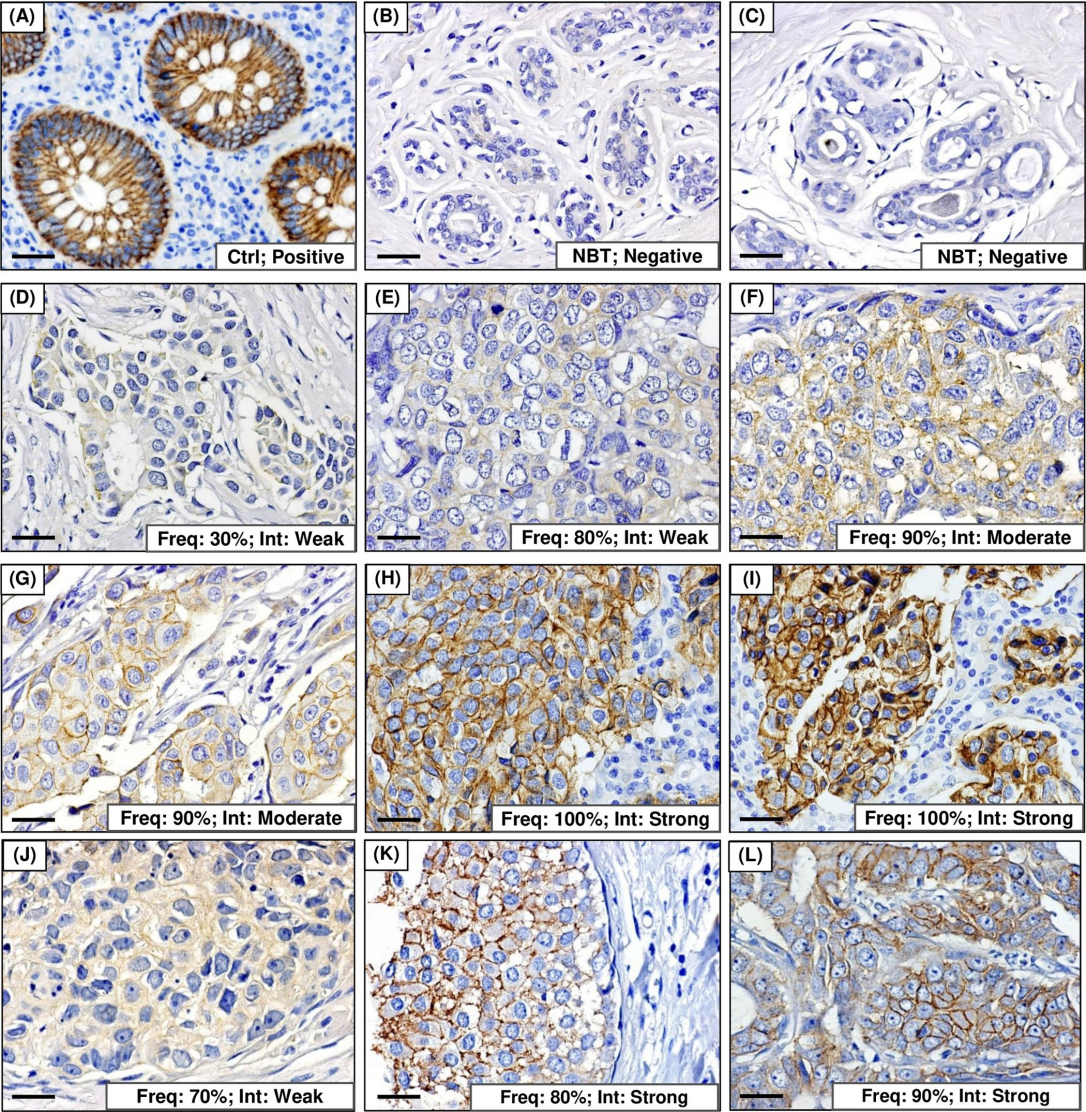
IHC of TRPM4 in normal breast tissues and breast cancer cases. (A) TRPM4 expression in appendix as control tissue (Ctrl) for IHC. (B-C) TRPM4 expression in two representative cases of normal breast tissues adjacent to tumor (NBT). (D-I) Representative breast cancer cases with various TRPM4 frequency and intensity. (J) TRPM4 expression in a representative fibroadenoma case. (K-L) Representative DCIS cases with 80–90% frequency and strong intensity. Freq: Frequency; Int: Intensity. All pictures were captured at 400x magnification. Scale bar for all images: 25 μm.

TRPM4 protein was expressed in 83 (83.8%) of the cases with frequency ranging from 10–100%, and median or mean frequency of 50% (Fig 3A). Majority of the breast cancer cases displayed weak TRPM4 intensity (n = 45/99; 45.5%) followed by moderate (n = 30/99; 30.3%) and strong (n = 8/99; 8.1%) intensity (Fig 3B). Half of the NBT cases (n = 5/10; 50%) were negative for TRPM4 (Fig 4B and 4C), and TRPM4 protein was significantly overexpressed in breast cancers (n = 83/99 TRPM4^+^; 83.8%) compared with normal breast ducts (n = 5/10 TRPM4^+^; 50%) (*p* = 0.022). Fig 4D–4I illustrate representative cases with TRPM4 frequency of 30%, 80%, 90% or 100%, and weak, moderate or strong intensity.

In addition, three cases in the TMA series were fibroadenomas where one of the cases was positive for TRPM4 (frequency: 50%; intensity: weak) (Fig 4J). All DCIS cases (n = 6) expressed TRPM4 with median and mean frequency of 50% and 60%, respectively, and weak (n = 3/6; 50%), moderate (n = 1/6; 16.7%) or strong (n = 2/6; 33.3%) intensity (Fig 4K and 4L).

### TRPM4 expression is associated with higher lymph node status and cancer stage

In terms of TRPM4 expression association with clinico-demographical parameters, higher TRPM4 frequency (median cut-off) was significantly associated with lymph node involvement (N1-N2 vs N0; *p* = 0.024) and higher stage (IIb-IIIb vs I-IIa; *p* = 0.005) (Table 1). No significant association was observed for other characteristics *i*.*e*. median age, tumor size, and cancer grade. Of note, only two breast cancer cases presented with grade 1, thus grades 1 and 2 (n = 60) were grouped for further analysis. For TRPM4 intensity, cases with negative or weak TRPM4 expression were grouped together to be analyzed against moderate or strong TRPM4 intensity due to the relatively smaller number of cases with strong (n = 8) or negative (n = 16) for TRPM4 expression. TRPM4 intensities (negative/weak vs moderate/strong intensities) were not associated with any of the clinico-demographical parameters investigated (Table 1).

**Table 1:** Association of TRPM4 protein expression with clinico-demographical and pathological parameters of breast cancer patients (n = 99). *p*<0.05 shown in bold.

Characteristics	n (%)	TRPM4 frequency			TRPM4 intensity		
		<Median (<50%)	≥Median (≥50%)	*p*-value	Negative/Weak	Moderate/Strong	*p*-value
**Age (years)**							
Median (range)	50 (27–81)						
<50	44 (44.4)	19 (43.2)	25 (56.8)	0.890	29 (65.9)	15 (34.1)	0.432
≥50	55 (55.6)	23 (41.8)	32 (58.2)		32 (58.2)	23 (41.8)	
**Tumor size**				0.445			
T1-T2	69 (69.7)	31 (44.9)	38 (55.1)		43 (62.3)	26 (37.7)	0.827
T3-T4	30 (30.3)	11 (36.7)	19 (63.3)		18 (60.0)	12 (40.0)	
**Lymph node status**							
N0	68 (68.7)	34 (50.0)	34 (50.0)	**0.024**	44 (64.7)	24 (35.3)	0.349
N1-N2	31 (21.3)	8 (25.8)	23 (74.2)		17 (54.8)	14 (45.2)	
Grade*							
1–2	62 (64.6)	22 (35.5)	40 (64.5)	0.097	37 (59.7)	25 (40.3)	0.841
3	34 (35.4)	18 (52.9)	16 (47.1)		21 (61.8)	13 (38.2)	
**Stage**^†^				**0.005**			
I-IIa	27 (67.5)	19 (70.4)	8 (29.6)		21 (77.8)	6 (22.2)	0.451 (F)
IIb-IIIb	13 (32.5)	3 (23.1)	10 (76.9)		8 (61.5)	5 (38.5)	
**ER**^‡^							
Negative	60 (61.2)	27 (45.0)	33 (55.0)	0.425	35 (58.3)	25 (41.7)	0.460
Positive	38 (38.8)	14 (36.8)	24 (63.2)		25 (65.8)	13 (34.2)	
**PR**^‡^							
Negative	57 (58.2)	28 (49.1)	29 (50.9)	0.085	36 (63.2)	21 (36.8)	0.643
Positive	41 (41.8)	13 (31.7)	28 (68.3)		24 (58.5)	17 (41.5)	
**HER2**^‡^							
Negative	67 (68.4)	32 (47.8)	35 (52.2)	0.080	40 (59.7)	27 (40.3)	0.649
Positive	31 (31.6)	9 (29.0)	22 (71)		20 (64.5)	11 (35.5)	
**Subtype**^‡^							
Luminal A	36 (36.7)	13 (36.1)	23 (63.9)	0.060	20 (55.6)	16 (44.4)	0.520
Luminal B	10 (10.2)	3 (30.0)	7 (70.0)		8 (80.0)	2 (20.0)	
HER2-enriched	21 (21.4)	6 (28.6)	15 (71.4)		12 (57.1)	9 (42.9)	
TNBC	31 (31.7)	19 (61.3)	12 (38.7)		20 (64.5)	11 (35.5)	
**Ki-67**^‡^							
<Median (<30%)	27 (45.8)	11 (40.7)	16 (59.3)	0.117	15 (55.6)	12 (44.4)	0.852
≥Median (≥30%)	32 (54.2)	7 (21.9)	25 (78.1)		17 (53.1)	15 (46.9)	

*Number of cases with available grade data: n = 96. Only two cases with grade 1, thus grades 1 and 2 were grouped;

**^†^**Number of cases with available stage data: n = 40.

**^‡^**One case without ER, PR or HER2 data; Number of cases with available Ki-67 staining frequency data: n = 59.

Additionally, both TRPM4 frequency (median cut-off) and intensity (negative/weak vs moderate/strong) were not associated with all pathological parameters investigated *i*.*e*. estrogen receptor (ER), progesterone receptor (PR), human growth factor receptor-2 (HER2), breast cancer subtype and Ki-67 (Table 1). Analysis without grouping TRPM4 intensities whereby each TRPM4 intensity (*i*.*e*. negative, weak, moderate, or strong individually without any grouping) was analyzed separately for each parameter also did not yield any significance for clinico-demographical parameters (S1 Table) and pathological markers (S2 Table). However, a trend toward PR (*p* = 0.085) or HER2 (*p* = 0.080) positivity was observed for cases with higher TRPM4 frequency (Table 1). Higher TRPM4 expression showed a non-significant trend for non-triple negative breast cancer (TNBC) subtypes including luminal A, luminal B and HER2-enriched (*p* = 0.060).

Associations of *TRPM4* transcript levels (median cut-off) with clinico-demographical and pathological parameters were also examined in the TCGA dataset of breast cancer cases (n = 500). Comparable with the observations in the TMA series, higher *TRPM4* expression (*TRPM4*^hi^) demonstrated a trend toward higher lymph node status (N1-N2 vs N0; *p* = 0.066), while significantly associated with PR positivity (*p* = 0.010) and non-TNBC subtypes including luminal A, luminal B and HER2-enriched (*p*<0.001) (Table 2). In addition, *TRPM4*^hi^ patients were significantly associated with ER positivity (*p*<0.001), while not associated with HER2 status and breast cancer stage. Of note, the proportion of *TRPM4*^hi^ patients with worse stage (IIb-IV; 54.2%) was higher than with lower stage (I-IIa; 44.6%) albeit it did not reach statistical significance (*p* = 0.102).

**Table 2:** Association of *TRPM4* mRNA expression with clinico-demographical and pathological parameters of TCGA breast cancer patients (n = 500). *p*<0.05 shown in bold.

Characteristics	n (%)	*TRPM4* expression (z-score)		
		<Median (<0.114)	≥Median (≥0.114)	*p*-value
**Age (years)**				
Median (range)	58 (27–81)			
<58	249 (49.8)	124 (49.8)	125 (50.2)	0.929
≥58	251 (50.2)	126 (50.2)	125 (49.8)	
**Lymph node status**				0.066
N0	251 (50.3)	136 (54.2)	115 (45.8)	
N1-N2	248 (49.7)	114 (46.0)	134 (54.0)	
**Stage**^**†**^				
I-IIa	222 (67.5)	123 (55.4)	99 (44.6)	0.102
IIb-IIIb	107 (32.5)	49 (45.8)	58 (54.2)	
**ER**				
Negative	115 (23.3)	79 (68.7)	36 (31.3)	**<0.001**
Positive	378 (76.7)	167 (44.2)	211 (55.8)	
**PR**				**0.010**
Negative	170 (34.6)	99 (58.2)	71 (41.8)	
Positive	322 (65.4)	148 (46.0)	174 (54.0)	
**HER2**				
Negative	409 (85.2)	214 (52.3)	195 (47.7)	0.450
Positive	71 (14.8)	28 (39.4)	43 (60.6)	
**Subtype**				
Luminal A	221 (45.6)	95 (43.0)	126 (57.0)	**<0.001**
Luminal B	112 (23.1)	55 (49.1)	57 (50.9)	
HER2-enriched	57 (11.7)	19 (33.3)	38 (66.7)	
TNBC	95 (19.6)	73 (76.8)	22 (23.2)	

### *TRPM4* transcript expression is associated with estrogen response and EMT gene sets in breast cancer

**Fig 5:**
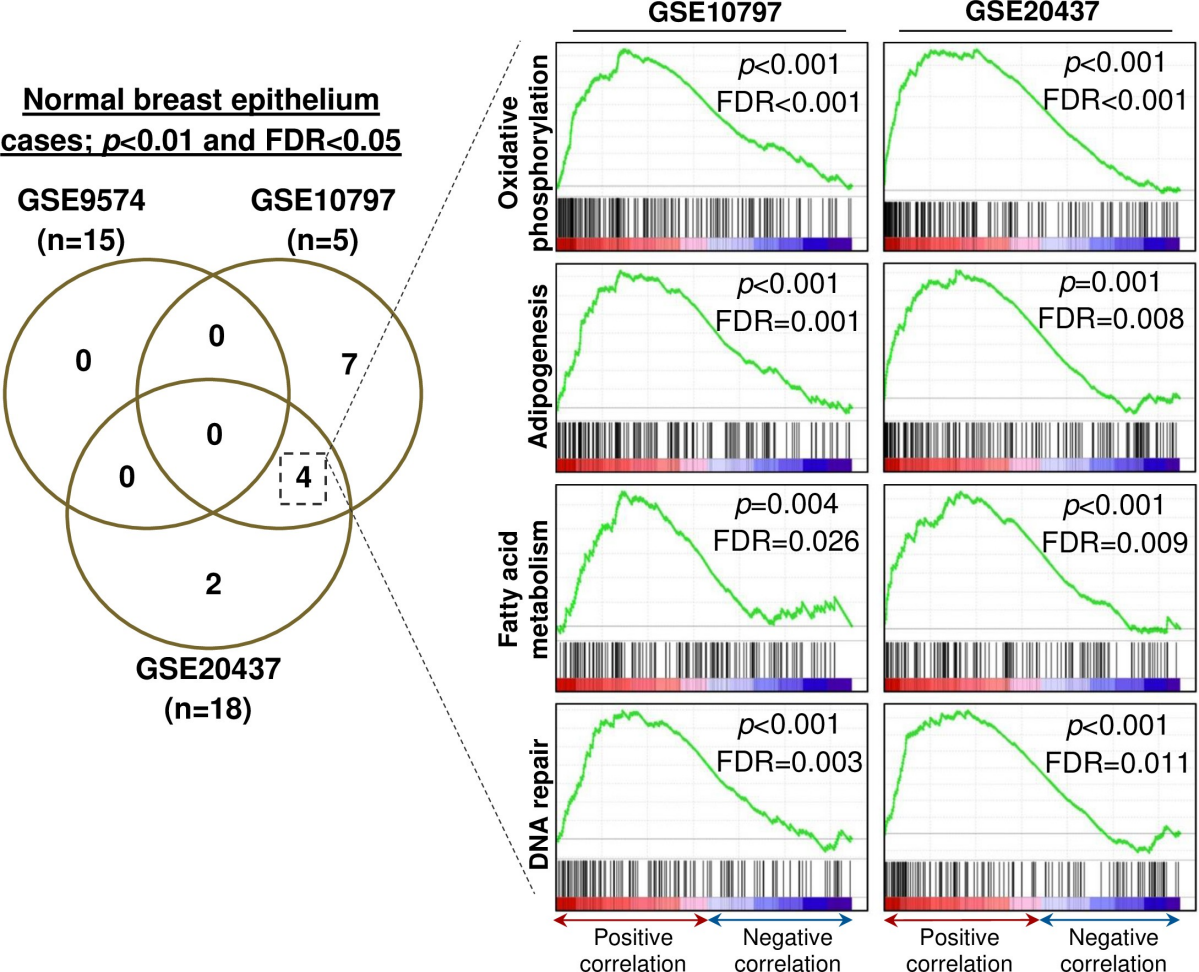
Consensus gene sets (Hallmark collection of the MSigDB) enriched according to *TRPM4* expression (219360_s_at) in three independent normal breast epithelium datasets (GSE9574, GSE10797, GSE20437).

We aimed to examine the functional relevance of TRPM4 in normal breast epithelium and breast cancer cases (three GEP datasets for each group) by associating the gene sets (Hallmark collection of the MSigDB) enriched according to *TRPM4* transcript expression through GSEA. Gene sets positively associated with *TRPM4* expression with *p*<0.01 and False Discovery Rate (FDR) of <0.05 were included for further analysis with a three-way Venn diagram comparison. In normal breast epithelium of reduction mammoplasty individuals (n = 38) derived from three independent GEP datasets, no consensus gene set was found in all three datasets according to *TRPM4* expression. Nonetheless, four gene sets were enriched in two of the GEP datasets (*i*.*e*. GSE10797 n = 5, and GSE20437 n = 18) consisting of Oxidative Phosphorylation (Hallmark ID: M5936), Adipogenesis (M5905), Fatty Acid Metabolism (M5935) and DNA Repair (M5898) gene sets (Fig 5). The list of consensus genes contributing to the enrichment of these gene sets in both GEP datasets is listed in S3 Table.

**Fig 6:**
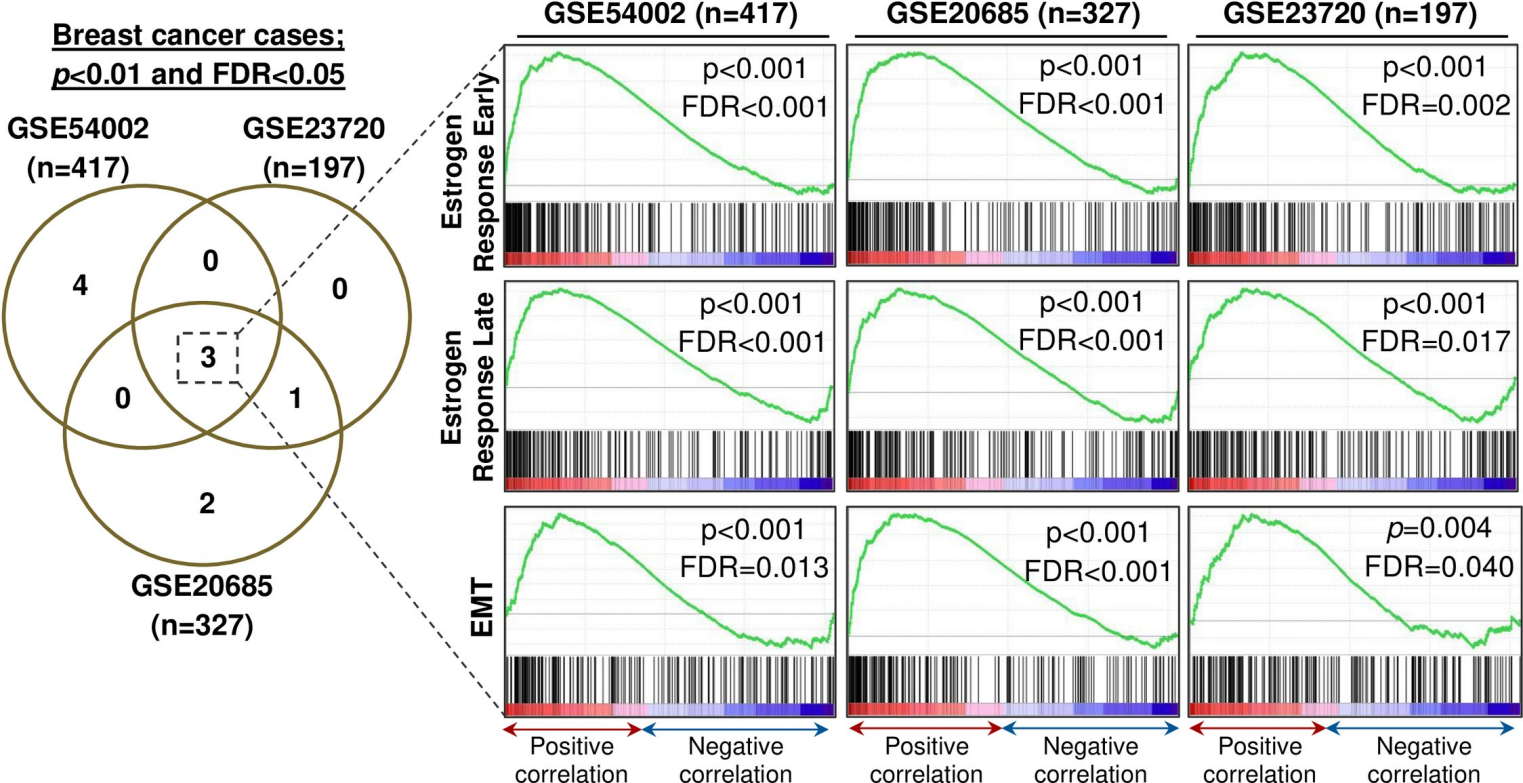
Consensus gene sets (Hallmark collection of the MSigDB) enriched according to *TRPM4* expression (219360_s_at) in three independent breast cancer datasets (GSE54002, GSE23720, GSE20685).

In breast cancer cases (n = 941) derived from three GEP datasets, three consensus gene sets were enriched according to *TRPM4* expression in all three GEP datasets (*i*.*e*. GSE54002 n = 417, GSE20685 n = 327, and GSE23720 n = 197) as follows: (1) Estrogen Response Early (Hallmark ID: M5906) gene set containing genes involved in early response to estrogen; (2) Estrogen Response Late (Hallmark ID: M5907) gene set containing genes involved in late response to estrogen; (3) Epithelial-Mesenchymal Transition (Hallmark ID: M5930) gene set containing genes involved in EMT (Fig 6). In particular, these gene sets were not enriched in normal breast epithelium cases, and none of the enriched gene sets in normal breast cases were enriched in breast cancers.

One consensus gene set positively associated with *TRPM4* transcript expression was shared in GSE20685 (n = 327) and GSE23720 (n = 197) series of breast cancer cases as demonstrated in the Venn diagram of Fig 6. The gene set was Myogenesis (ID: M5909) containing genes involved in the development of skeletal muscle. The GSEA graphs are shown in S1 Fig and the list of genes that contributed to the core enrichment of the gene set in both GEP datasets is shown in S5 Table.

**Fig 7:**
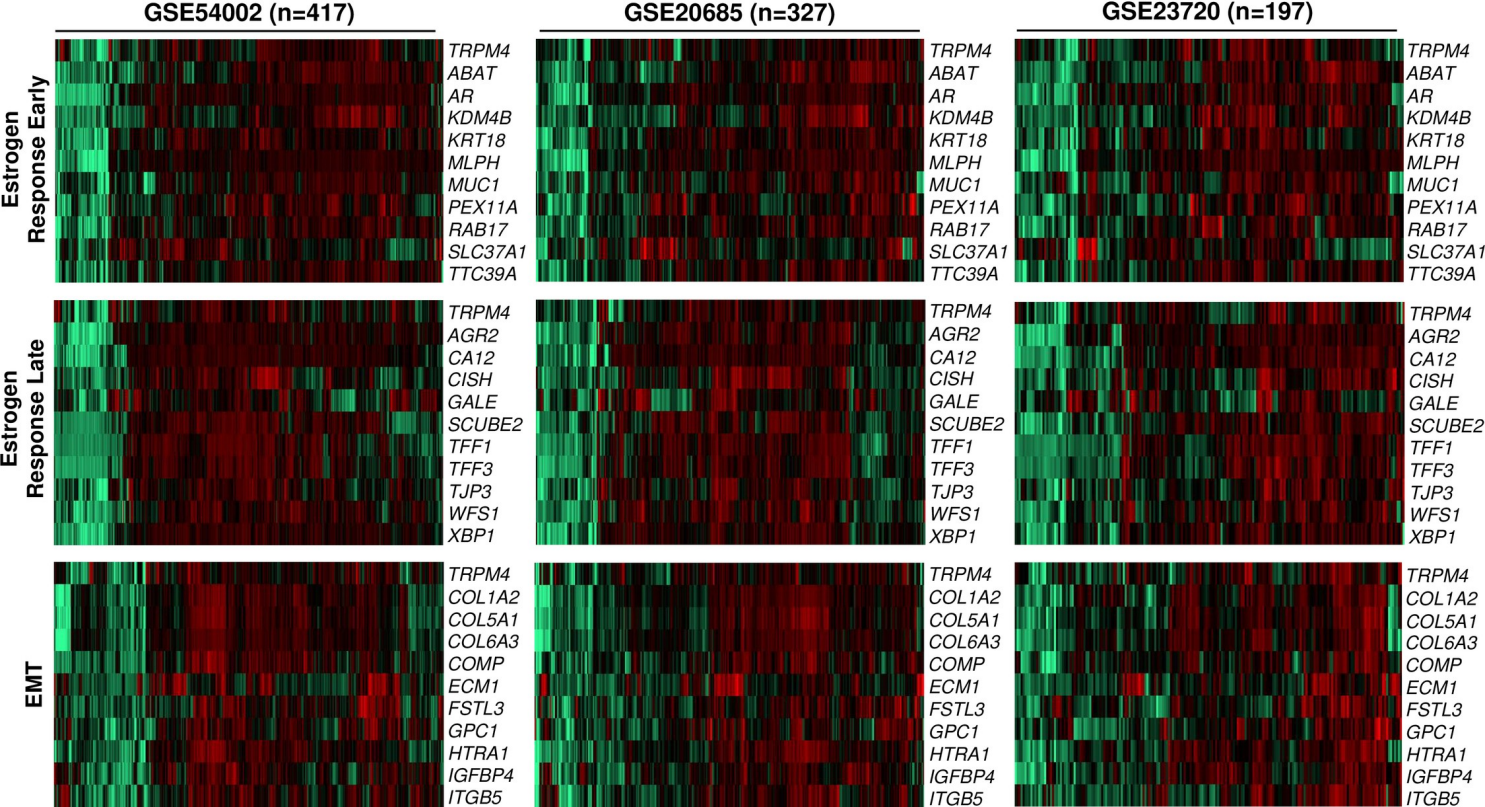
Heat map of *TRPM4* and top representative genes (n = 10 in each gene set and GEP dataset) contributing to the core enrichment of each gene set.

A total of 57, 43, and 31 genes contributed to the core enrichment across all three GEP breast cancer datasets for Estrogen Response Early, Estrogen Response Late, and EMT gene set, respectively (S4 Table). From these consensus genes across the three GEP datasets, 10 genes were shortlisted to represent each gene set for illustration in heat maps as follows (Fig 7): (1) Estrogen Response Early (*ABAT*, *AR*, *KDM4B*, *KRT18*, *MLPH*, *MUC1*, *PEX11A*, *RAB17*, *SLC37A1* and *TTC39A*); (2) Estrogen Response Late (*AGR2*, *CA12*, *CISH*, *GALE*, *SCUBE2*, *TFF1*, *TFF3*, *TJP3*, *WFS1* and XBP1); (3) Epithelial-Mesenchymal Transition (*COL1A2*, *COL5A1*, *COL6A3*, *COMP*, *ECM1*, *FSTL3*, *GPC1*, *HTRA1*, *IGFBP4* and *ITGB5*).

Androgen receptor (AR) is an emerging therapeutic target in breast cancer where it is expressed in majority (60–80%) of breast cancers with higher prevalence in ER-α^+^ tumors [45]. *AR* was one of the genes that contributed to the enrichment of the estrogen response gene sets positively associated with *TRPM4* expression as demonstrated above. To examine the potential association between TRPM4 and AR further, the IHC data of TRPM4 and AR in breast cancer cases were obtained from Human Protein Atlas (HPA) database (https://www.proteinatlas.org/) [46]. Both the anti-TRPM4 (HPA041169) and anti-AR (CAB065764) antibodies used for IHC received the “Enhanced” validation score by HPA whereby IHC staining with the antibodies corresponded with mRNA expression levels across 37 normal tissues by HPA. In HPA’s breast cancer cases, TRPM4 and AR protein was expressed in 90.9% (n = 10/11) and 100% (n = 12/12) of the cases, respectively. Four cases contained the IHC staining images of both proteins and visualization of the IHC staining images of these four cases suggested that TRPM4 and AR had similar expression profile in breast cancer cells (S2 Fig). The frequency and intensity of each protein in these four cases, as annotated by HPA, were partially similar (S6 Table) as follows: Case #1: TRPM4 >75% (frequency), moderate (intensity); AR >75%, moderate; Case #2: TRPM4 >75%, moderate; AR 25–75%, weak; Case #3: TRPM4 <25%, weak; AR >75%, moderate; Case #4: TRPM4 negative; AR <25%, moderate.

## Discussion

In this study, we showed that TRPM4 protein was overexpressed in breast cancers compared with normal breast epithelial ducts. This observation is comparable with TRPM4 expression in other solid tumors versus their counterpart non-malignant tissues. In prostate cancer, TRPM4 staining intensity was significantly higher in prostate cancer cases than benign or non-malignant prostate tissues as well as stromal cells of prostate glands [17, 18]. TRPM4 was more intensely expressed in tumor buds of colorectal cancer than normal ducts of non-malignant colorectal tissues [24]. *TRPM4* transcript was also overexpressed in cervical cancer cases compared with normal cervical epithelium samples [23].

Breast cancer evolves from normal epithelial ducts through a sequence of increasingly abnormal proliferative lesions that begin with atypical hyperplasia, to pre-malignant in situ disease, before progressing into neoplasia [47, 48]. DCIS is characterized by epithelial carcinoma within the ducts, surrounded by myoepithelial cells and with the basement membrane intact [49]. Patients diagnosed with DCIS have a high risk for subsequent development into invasive carcinoma particularly if left untreated [49, 50]. We observed that TRPM4 was expressed in half a proportion of normal breast ducts but expressed in all DCIS cases with similar frequency and intensity distribution as breast cancer cases. This suggests a progressive pattern of TRPM4 expression upregulation from normal into pre-cancerous DCIS, and its expression might be maintained in DCIS that develops into invasive breast carcinoma. However, larger number of DCIS cases complemented by TRPM4 functional studies is required to conclusively prove this.

TRPM4 expression was associated with advanced clinical parameters such as higher lymph node status (N1 and N2) and cancer grade (IIb and IIIb) in the TMA breast cancer series. This is suggestive of the causative roles of TRPM4 in breast cancer metastasis and invasion into axillary lymph nodes. TRPM4 has been frequently implicated in triggering the migration of various types of immune cells including dendritic cells [12], T helper type 1 (Th1) cells [13] and mast cells [51]. In malignancies, multiple studies have demonstrated the requirement of TRPM4 for the migration and invasion of prostate cancer cells [17, 20, 21]. Moreover, colorectal cancer cell clones with TRPM4 knockout showed decreased migration and invasion [24].

One of the key mechanisms that triggers cancer invasion and metastasis is through activation of EMT. *TRPM4* transcript expression was associated with EMT gene set in breast cancer cases but not in normal breast epithelium cases as demonstrated in this study, indicating its potential involvement in triggering EMT for breast cancer cells metastasis. This observation is in line with those observed in other cancers pertaining to TRPM4 and EMT as follows: (1) Independent studies have shown that TRPM4 knockdown could suppress migration and invasion of prostate cancer cells through reduction of EMT. Knockdown of TRPM4 correlated with reduction of mesenchymal markers including N-cadherin and vimentin, while expression of epithelial markers was increased such as E-cadherin and Snail [20, 21]; (2) In colorectal cancer, tumor budding is characterized by presence of disseminated colorectal cancer cells driven by EMT for metastasis [52–54]. High TRPM4 protein intensity was associated with increased number of tumor buds and infiltrative growth pattern in colorectal cancer patient cases, and late-stage metastatic colorectal cancer cell lines had higher TRPM4 protein expression [24]; (3) *TRPM4* transcript expression was positively associated with EMT genes of adhesion molecules or extracellular matrix origin including collagens (*e*.*g*. *COL1A2*, *COL5A1*, *COL6A3*) and extracellular matrix 1 (*ECM1*) in our GSEA results. Collagen proteins promote metastasis of breast cancer cells [55, 56], while ECM1 is a key player in triggering EMT and metastasis of breast cancer cells through stabilization of β-catenin [57, 58]. The key roles of TRPM4 in activating the β-catenin signaling pathway for the EMT and invasion of prostate cancer cells [19–21] might also occur in breast cancer to activate EMT for metastasis that requires further investigations.

Breast cancer can be subtyped based on the expression of surface receptors including ER, PR and HER2 [59]. Approximately two-thirds of breast cancer cases are ER-α^+^ which can be controlled by modulators of ER-α such as tamoxifen [60]. However, tamoxifen resistance development is common, hence novel therapies targeting ER-α is required. Our GSEA results showed that *TRPM4* transcript was positively-associated with estrogen response gene sets, suggesting its involvement in ER signaling pathway. In particular, *AR* was one of the top genes positively-associated with *TRPM4* transcript expression in the enriched estrogen response gene sets.

AR is frequently expressed in breast cancers and proposed to be a target in the disease [45]. AR competes with ER at the level of ER elements that impairs ER-dependent gene transcription [61], and AR has been proposed as a therapeutic target in ER-α^─^ breast cancers that retain AR expression [62, 63]. Circulating androgens are present at physiological conditions in females with changing levels during life, and high levels of circulating androgens are associated with increased risk of breast cancer development [64]. In prostate cancer, the tumors are dependent on AR and androgen-deprivation therapy is a gold standard therapy in advanced prostate cancer [45]. Interestingly, TRPM4 is highly expressed in androgen-sensitive prostate cancers required for their aggressive phenotypes [19, 20]. Moreover, AR is expressed in all grades of breast DCIS that confers unfavorable prognosis [65–67], and TRPM4 was also expressed in all DCIS cases examined in this study. The similarities in the expression profile of TRPM4 and AR in DCIS, breast and prostate cancers suggest a connection of TRPM4 with AR signaling pathway in tumors, and this warrants future investigations.

A recent study reported that K^+^ channel tetramerization domain 5 (KCTD5) is a positive regulator of TRPM4 activity whereby KCTD5 promotes cell migration and contractility through regulation of TRPM4 [68]. Essentially, in a series of normal breast tissue samples (n = 5) and breast cancer cases (n = 43), the authors demonstrated that *TRPM4* mRNA expression was significantly higher in breast cancers or patients with higher breast cancer stage, comparable with the observations in our study. It was also reported that *TRPM4* transcript expression was significantly upregulated in TNBC cases compared with normal breast tissues, in contrast with our observations in TCGA and TMA series of breast cancer cases in which TRPM4 was significantly less common in TNBC cases compared with other breast cancer subtypes (luminal A, luminal B, or HER2-enriched). We recommend that validation at the protein level in larger series of breast cancer cases is required to resolve this, as well as to validate the association of TRPM4 with AR protein expression.

Nevertheless, TRPM4 is listed as one of the druggable genes in the redefined list of the druggable genome [69], and it is shortlisted as one of the potentially druggable proteins of the human proteome by the Human Protein Atlas consortium [46]. TRPM4 thus represents a potential therapeutic target in breast cancer. Small molecule inhibitors of TRPM4 include the most commonly investigated TRPM4 inhibitor 9-phenanthrol (a phenanthrene derivative) [70] but the compound lacks specificity as it also targets the Ca^2+^-activated Cl^-^ channel TMEM16A in arterial myocytes [71], or the recently identified specific and potent TRPM4 inhibitor aryloxyacyl-anthranilic 5 (termed as compound 5) with approximately 20 times stronger TRPM4 inhibition than 9-phenanthrol [72]. A recent TRPM4 blocking antibody has been generated termed as M4P that demonstrates specificity for TRPM4 without binding to TRPM5 channel. M4P is capable of binding to TRPM4 in ischemic stroke and ameliorating reperfusion injury by improving blood-brain barrier integrity in a rat model of stroke reperfusion [73]. Hence, TRPM4 blocking antibody expands the option of TRPM4 blockers not limited to small molecule inhibitors. Furthermore, the atomic-level structure of TRPM4 has been revealed recently through cryo-electron microscopy by independent groups. The transmembrane domain of TRPM4 contains a Ca^2+^ binding site, and ATP binds its N-terminal nucleotide-binding domain (NBD) that subsequently inhibits activities of TRPM4 [74–76]. These atomic-level maps of TRPM4 facilitates ongoing development of specific TRPM4 inhibitors that can act through selectively obstructing its Ca^2+^-activation binding site or to inhibit TRPM4 via NBD binding, providing expanded avenues to therapeutically target TRPM4 in cancers.

In conclusion, our study demonstrated frequent TRPM4 expression in breast cancer associated with poorer clinical parameters, and that its expression was associated with ER signaling and EMT. These findings support future experimental investigations on TRPM4 inhibitors in the destruction of breast cancer cells, and their potential inhibitory effects on ER signaling cascade and EMT phenotypes.

## Supporting information

S1 Fig GSEA graphs of the Myogenesis (ID: M5909) gene set positively associated with *TRPM4* transcript expression (*p*<0.001 and FDR<0.05) in two GEP datasets of breast cancer cases, GSE20685 (n = 327) and GSE23720 (n = 197). (TIF)

S2 Fig Four breast cancer cases (patient ID 1874, 1910, 2805, 2160) of TRPM4 (HPA041169 antibody) and AR (CAB065764 antibody) IHC staining obtained from Human Protein Atlas database. (PDF)

S1 Table Association of each TRPM4 intensity (negative, weak, moderate or strong) with clinico-demographical parameters of breast cancer patients (n = 99). (DOCX)

S2 Table Association of each TRPM4 intensity (negative, weak, moderate or strong) with pathological parameters of breast cancer patients (n = 99). (DOCX)

S3 Table List of consensus genes contributing to the enrichment of Oxidative Phosphorylation (Hallmark ID: M5936), Adipogenesis (M5905), Fatty Acid Metabolism (M5935) and DNA Repair (M5898) gene sets according to *TRPM4* expression in normal breast tissues datasets (GEO ID: GSE10797 and GSE20437). (DOCX)

S4 Table List of consensus genes contributing to the enrichment of estrogen responses (Hallmark ID: M5906 and M5907) and EMT gene sets (M5930) according to *TRPM4* expression in breast cancer datasets (GEO ID: GSE54002, GSE20685 and GSE23720). (DOCX)

S5 Table List of consensus genes contributing to the enrichment of Myogenesis gene set (Hallmark ID: M5909) according to *TRPM4* expression in breast cancer datasets (GEO ID: GSE20685 and GSE23720). (DOCX)

S6 Table IHC of TRPM4 or AR in breast cancer cases according to Human Protein Atlas database. (DOCX)
