# Supplementary material for: TRPM4 is overexpressed in breast cancer associated with estrogen response and epithelial-mesenchymal transition gene sets
Source: PLoS One. 2020 Jun 2;15(6):e0233884. doi: 10.1371/journal.pone.0233884 (PMC7266295; doi:10.1371/journal.pone.0233884)
Supplement: S2 Fig — (PDF) [file pone.0233884.s002.pdf]

S2 Fig

(A)

Patient ID: 1874

TRPM4

Frequency: >75%

Intensity: Moderate

AR

Frequency: >75%

Intensity: Moderate

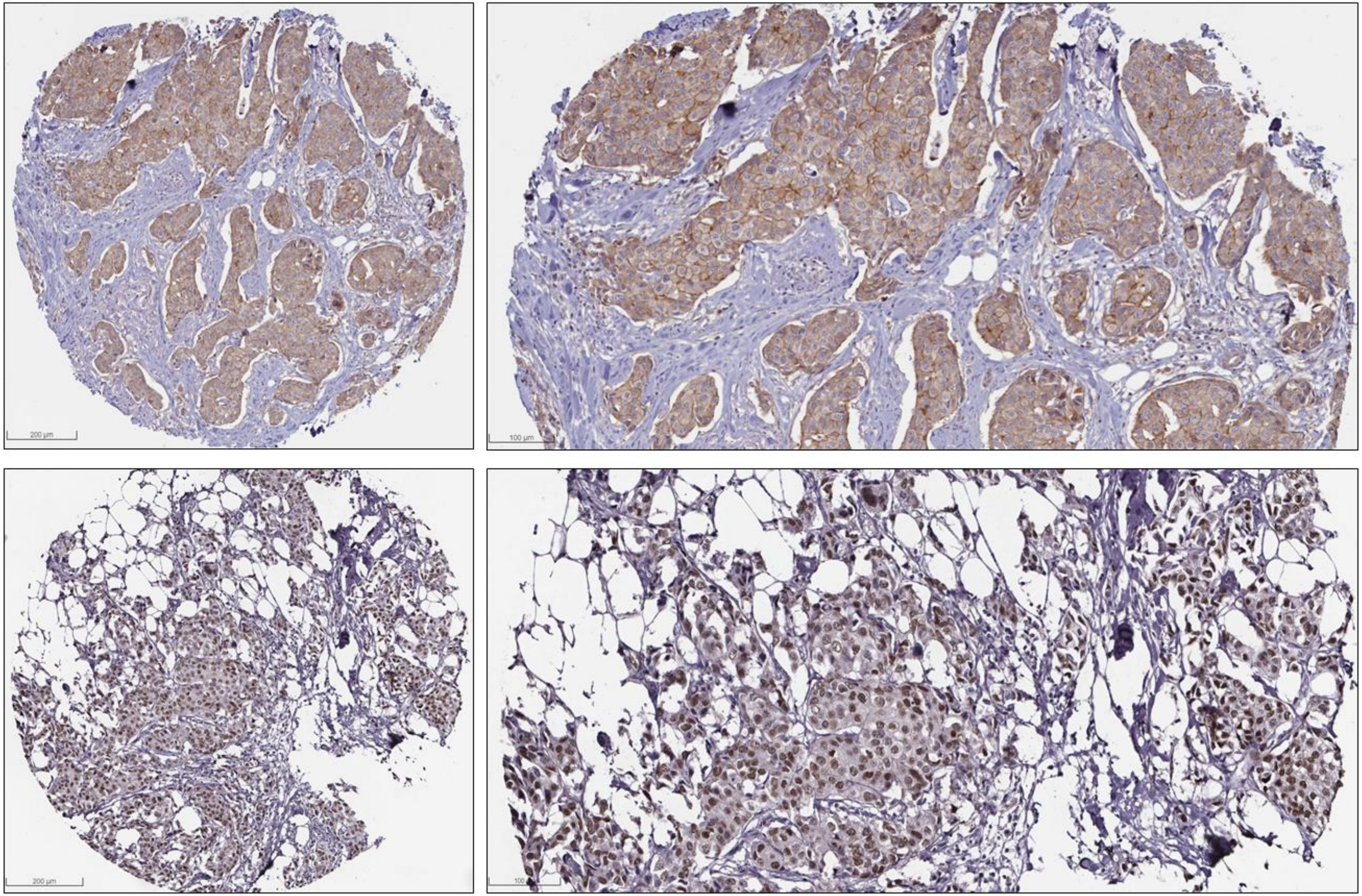

(B)

Patient ID: 1910

TRPM4

Frequency: >75%

Intensity: Moderate

AR

Frequency: 25-75%

Intensity: Weak

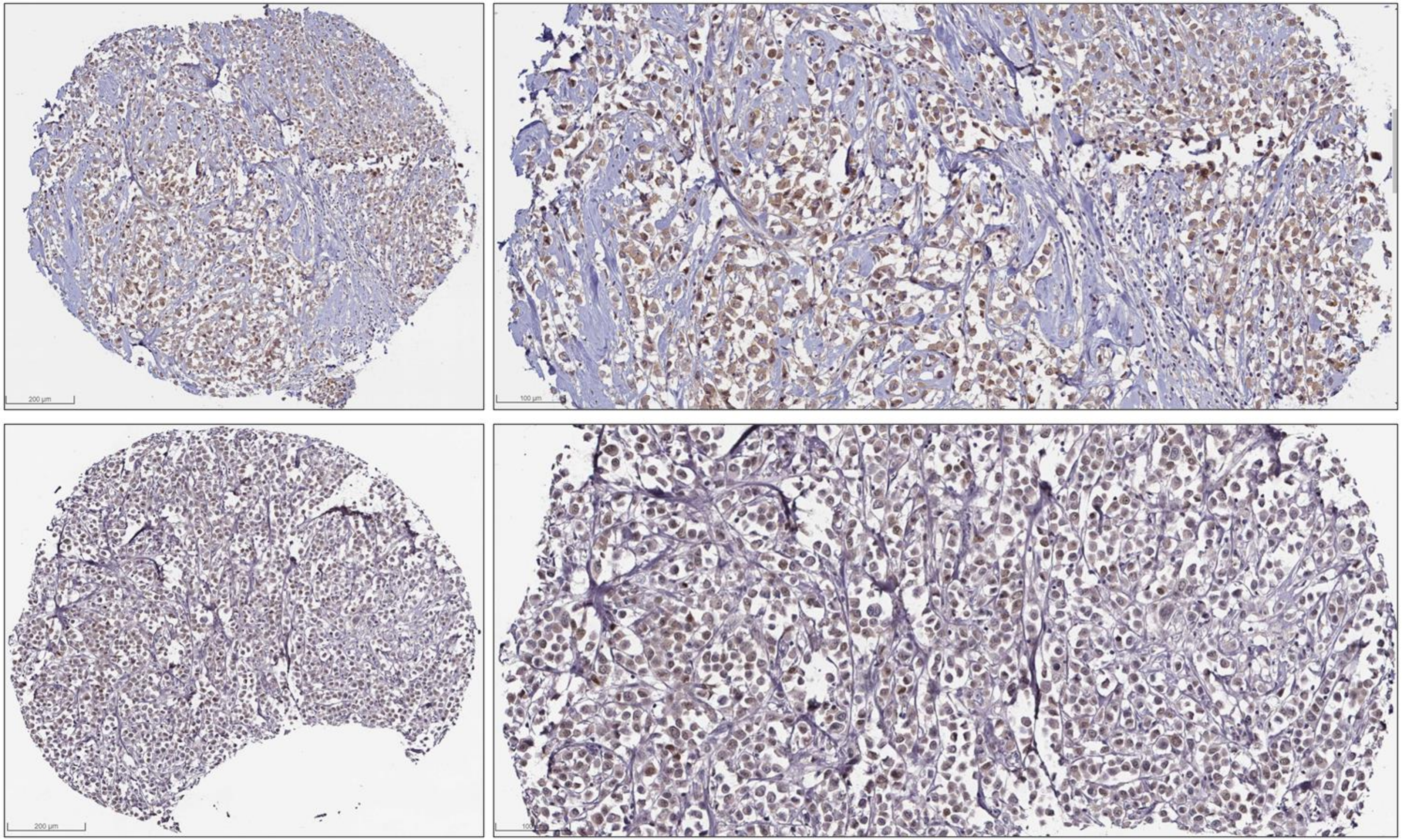

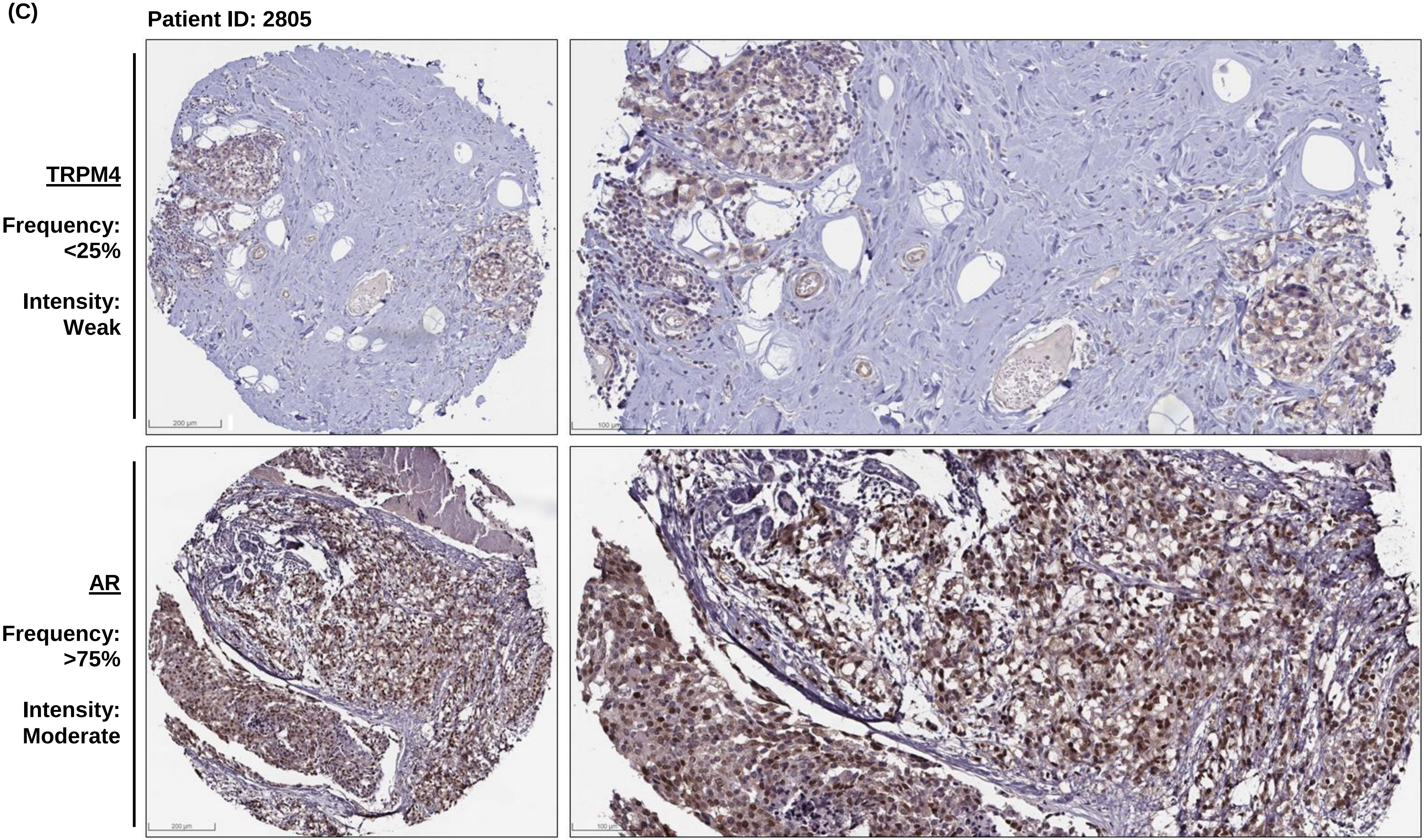
(C)
Patient ID: 2805
TRPM4
Frequency: <25%
Intensity: Weak
AR
Frequency: >75%
Intensity: Moderate
200 µm
100 µm
200 µm
100 µm

(D)

Patient ID: 2160

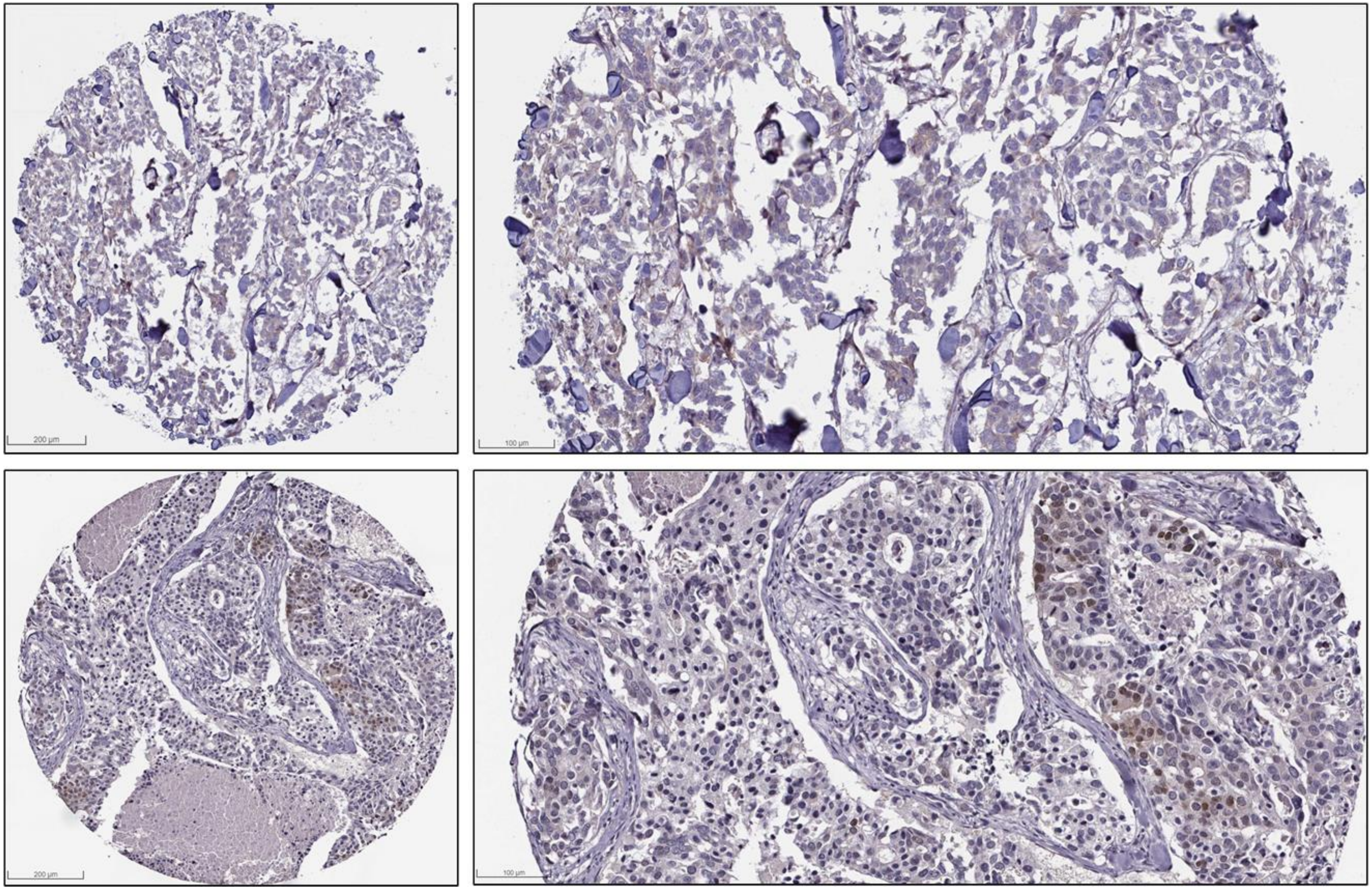

TRPM4

Frequency: Negative

Intensity: Negative

AR

Frequency: <25%

Intensity: Moderate
