# Supplementary material for: TRPM4 is overexpressed in breast cancer associated with estrogen response and epithelial-mesenchymal transition gene sets
Source: PLoS One. 2020 Jun 2;15(6):e0233884. doi: 10.1371/journal.pone.0233884 (PMC7266295; doi:10.1371/journal.pone.0233884)
Supplement: S1 Table — (DOCX) [file pone.0233884.s003.docx]

**S1 Table. Association of each TRPM4 intensity separately (negative, weak, moderate or strong) with clinico-demographical parameters of breast cancer patients (n=99).**

| **Characteristics** | **n (%)** | **TRPM4 Intensity** | | | | |
| --- | --- | --- | --- | --- | --- | --- |
|  |  | **Negative** | **Weak** | **Mode-rate** | **Strong** | ***p*-value** |
| **Age (years)** |  |  |  |  |  |  |
| Median (range) | 99 (27-81) |  |  |  |  |  |
| <50 | 44 (44.4) | 10 (10.1) | 19 (19.2) | 12 (12.1) | 3 (3.0) | 0.477 (F) |
| ≥50 | 55 (55.6) | 6 (6.1) | 26 (26.3) | 18 (18.2) | 5 (5.0) |  |
| **Tumor size** | |  |  |  |  |  |
| T1-T2 | 69 (69.7) | 10 (10.1) | 33 (33.3) | 19 (19.2) | 7 (7.1) | 0.525 (F) |
| T3-T4 | 30 (30.3) | 6 (6.1) | 12 (12.1) | 11 (11.1) | 1 (1.0) |  |
| **Lymph node status** | |  |  |  |  |  |
| N0 | 68 (68.7) | 13 (13.1) | 31 (31.3) | 21 (21.2) | 3 (3.0) | 0.186 |
| N1-N2 | 31 (21.3) | 3 (3.0) | 14 (14.1) | 9 (9.1) | 5 (5.1) |  |
| **Grade*** | |  |  |  |  |  |
| 1-2 | 62 (64.6) | 8 (8.3) | 29 (30.2) | 18 (18.8) | 7 (7.3) | 0.377 |
| 3 | 34 (35.4) | 7 (7.3) | 14 (14.6) | 12 (12.5) | 1 (1.0) |  |
| **Stage^†^** | |  |  |  |  |  |
| I-IIa | 27 (67.5) | 7 (17.5) | 14 (35.0) | 4 (10.0) | 2 (5.0) | 0.722 (F) |
| IIb-IIIb | 13 (32.5) | 2 (5.0) | 6 (15.0) | 4 (10.0) | 1 (2.5) |  |

*****Number of cases with available grade data: n=96; **^†^**Number of cases with available stage data: n=40; (F) denotes Fisher’s exact test. No significant association was observed for
each parameter.
