# Supplementary material for: TRPM4 is overexpressed in breast cancer associated with estrogen response and epithelial-mesenchymal transition gene sets
Source: PLoS One. 2020 Jun 2;15(6):e0233884. doi: 10.1371/journal.pone.0233884 (PMC7266295; doi:10.1371/journal.pone.0233884)
Supplement: S2 Table — (DOCX) [file pone.0233884.s004.docx]

**S2 Table. Association of each TRPM4 intensity separately (negative, weak, moderate or strong) with pathological parameters of breast cancer patients (n=99).**

| **Characteristics** | **n (%)** | **TRPM4 Intensity (n=99)** | | | | |
| --- | --- | --- | --- | --- | --- | --- |
|  |  | **Negative** | **Weak** | **Mode-rate** | **Strong** | ***p*-value** |
| **ER*** |  |  |  |  |  |  |
| Negative | 60 (61.2) | 11 (11.2) | 24 (24.5) | 19 (19.4) | 6 (6.1) | 0.639 (F) |
| Positive | 38 (38.8) | 5 (5.1) | 20 (20.4) | 11 (11.2) | 2 (2.1) |  |
| **PR*** |  |  |  |  |  |  |
| Negative | 57 (58.2) | 11 (11.2) | 25 (25.5) | 16 (16.3) | 5 (5.1) | 0.802 (F) |
| Positive | 41 (41.8) | 5 (5.1) | 19 (19.4) | 14 (14.3) | 3 (3.1) |  |
| **HER2*** |  |  |  |  |  |  |
| Negative | 67 (68.4) | 13 (13.3) | 27 (27.6) | 21 (21.4) | 6 (6.1) | 0.525 (F) |
| Positive | 31 (31.6) | 3 (3.1) | 17 (17.3) | 9 (9.2) | 2 (2.0) |  |
| **Subtype*** |  |  |  |  |  |  |
| Luminal 1 | 36 (36.7) | 5 (5.1) | 15 (15.3) | 13 (13.3) | 3 (3.1) | 0.803 (F) |
| Luminal 2 | 10 (10.2) | 1 (1.0) | 7 (7.1) | 2 (2.0) | 0 (0) |  |
| Non-luminal HER2^+^ | 21 (21.4) | 2 (2.0) | 10 (10.2) | 7 (7.1) | 2 (2.0) |  |
| TNBC | 31 (31.7) | 8 (8.2) | 12 (12.3) | 8 (8.2) | 3 (3.1) |  |
| **Ki-67** |  |  |  |  |  |  |
| <Median (<30%) | 27 (45.8) | 4 (6.8) | 11 (18.6) | 11 (18.6) | 1 (1.7) | 0.630 (F) |
| ≥Median (≥30%) | 32 (54.2) | 3 (5.1) | 14 (23.8) | 11 (18.6) | 4 (6.8) |  |

*****One case without ER, PR or HER2 data; **^†^**Number of cases with available Ki-67 staining frequency data: n=59. (F) denotes Fisher’s exact test. No significant association was observed for each parameter.
