# Supplementary material for: TRPM4 is overexpressed in breast cancer associated with estrogen response and epithelial-mesenchymal transition gene sets
Source: PLoS One. 2020 Jun 2;15(6):e0233884. doi: 10.1371/journal.pone.0233884 (PMC7266295; doi:10.1371/journal.pone.0233884)
Supplement: S3 Table — (DOCX) [file pone.0233884.s005.docx]

**S3 Table. List of consensus genes contributing to the enrichment of Oxidative Phosphorylation (Hallmark ID: M5936), Adipogenesis (M5905), Fatty Acid Metabolism (M5935) and DNA Repair (M5898) gene sets according to *TRPM4* expression in normal breast tissues datasets (GEO ID: GSE10797 and GSE20437).**

| **List of Oxidative Phosphorylation (ID: M5936) genes with core enrichment in 2 datasets (GSE10797 and GSE20437)** | |  |
| --- | --- | --- |
| **Gene** | **Name** |  |
| *ACAA1* | acetyl-CoA acyltransferase 1 |  |
| *ACADVL* | acyl-CoA dehydrogenase, very long chain |  |
| *ALAS1* | aminolevulinate, delta-, synthase 1 |  |
| *ATP6AP1* | ATPase, H^+^ transporting, lysosomal accessory protein 1 |  |
| *ATP6V0B* | ATPase, H^+^ transporting, lysosomal 21kDa, V0 subunit b |  |
| *ATP6V0C* | ATPase, H^+^ transporting, lysosomal 16kDa, V0 subunit c |  |
| *ATP6V1E1* | ATPase, H^+^ transporting, lysosomal 31kDa, V1 subunit E1 |  |
| *BCKDHA* | branched chain keto acid dehydrogenase E1, alpha polypeptide |  |
| *COX17* | COX17 cytochrome c oxidase assembly homolog (*S. cerevisiae*) |  |
| *COX4I1* | cytochrome c oxidase subunit IV isoform 1 |  |
| *COX5B* | cytochrome c oxidase subunit Vb |  |
| *COX6A1* | cytochrome c oxidase subunit VIa polypeptide 1 |  |
| *COX7A2* | cytochrome c oxidase subunit VIIa polypeptide 2 (liver) |  |
| *COX7C* | cytochrome c oxidase subunit VIIc |  |
| *CYB5R3* | cytochrome b5 reductase 3 |  |
| *ECHS1* | enoyl CoA hydratase, short chain, 1, mitochondrial |  |
| *ECI1* | enoyl-CoA delta isomerase 1 |  |
| *ETFB* | electron-transfer-flavoprotein, beta polypeptide |  |
| *FH* | fumarate hydratase |  |
| *GPX4* | glutathione peroxidase 4 |  |
| *HADHA* | hydroxyacyl-CoA dehydrogenase/3-ketoacyl-CoA thiolase/enoyl-CoA hydratase (trifunctional protein), alpha subunit |  |
| *HSD17B10* | hydroxysteroid (17-beta) dehydrogenase 10 |  |
| *IDH3B* | isocitrate dehydrogenase 3 (NAD^+^) beta |  |
| *IDH3G* | isocitrate dehydrogenase 3 (NAD^+^) gamma |  |
| *MDH2* | malate dehydrogenase 2, NAD (mitochondrial) |  |
| *MRPS12* | mitochondrial ribosomal protein S12 |  |
| *MRPS15* | mitochondrial ribosomal protein S15 |  |
| *NDUFA1* | NADH dehydrogenase (ubiquinone) 1 alpha subcomplex, 1, 7.5kDa |  |
| *NDUFA2* | NADH dehydrogenase (ubiquinone) 1 alpha subcomplex, 2, 8kDa |  |
| *NDUFA3* | NADH dehydrogenase (ubiquinone) 1 alpha subcomplex, 3, 9kDa |  |
| *NDUFA6* | NADH dehydrogenase (ubiquinone) 1 alpha subcomplex, 6, 14kDa |  |
| *NDUFA7* | NADH dehydrogenase (ubiquinone) 1 alpha subcomplex, 7, 14.5kDa |  |
| *NDUFA9* | NADH dehydrogenase (ubiquinone) 1 alpha subcomplex, 9, 39kDa |  |
| *NDUFAB1* | NADH dehydrogenase (ubiquinone) 1, alpha/beta subcomplex, 1, 8kDa |  |
| *NDUFB1* | NADH dehydrogenase (ubiquinone) 1 beta subcomplex, 1, 7kDa |  |
| *NDUFB2* | NADH dehydrogenase (ubiquinone) 1 beta subcomplex, 2, 8kDa |  |
| *NDUFB8* | NADH dehydrogenase (ubiquinone) 1 beta subcomplex, 8, 19kDa |  |
| *NDUFC1* | NADH dehydrogenase (ubiquinone) 1, subcomplex unknown, 1, 6kDa |  |
| *NDUFC2* | NDUFC2-KCTD14) NADH dehydrogenase (ubiquinone) 1, subcomplex unknown, 2, 14.5kDa |  |
| *NDUFS2* | NADH dehydrogenase (ubiquinone) Fe-S protein 2, 49kDa (NADH-coenzyme Q reductase) |  |
| *NDUFS6* | NADH dehydrogenase (ubiquinone) Fe-S protein 6, 13kDa (NADH-coenzyme Q reductase) |  |
| *NDUFV1* | NADH dehydrogenase (ubiquinone) flavoprotein 1, 51kDa |  |
| *PDHB* | pyruvate dehydrogenase (lipoamide) beta |  |
| *PHB2* | prohibitin 2 |  |
| *PHYH* | phytanoyl-CoA 2-hydroxylase |  |
| *POR* | P450 (cytochrome) oxidoreductase |  |
| *RETSAT* | retinol saturase (all-trans-retinol 13,14-reductase) |  |
| *SLC25A11* | solute carrier family 25 (mitochondrial carrier; oxoglutarate carrier), member 11 |  |
| *SLC25A12* | solute carrier family 25 (aspartate/glutamate carrier), member 12 |  |
| *SLC25A6* | solute carrier family 25 (mitochondrial carrier; adenine nucleotide translocator), member 6 |  |
| *SURF1* | surfeit 1 |  |
| *TCIRG1* | T-cell, immune regulator 1, ATPase, H^+^ transporting, lysosomal V0 subunit A3 |  |
| *TIMM13* | translocase of inner mitochondrial membrane 13 homolog (yeast) |  |
| *UQCR11* | ubiquinol-cytochrome c reductase, complex III subunit XI |  |
| *UQCRQ* | ubiquinol-cytochrome c reductase, complex III subunit VII, 9.5kDa |  |
| **List of Adipogenesis (M5905) genes with core enrichment in 2 datasets (GSE10797 and GSE20437)** | | |
| Gene | Name | |
| *AK2* | adenylate kinase 2 | |
| *ALDH2* | aldehyde dehydrogenase 2 family (mitochondrial) | |
| *ALDOA* | aldolase A, fructose-bisphosphate | |
| *ANGPTL4* | angiopoietin-like 4 | |
| *ARAF* | v-raf murine sarcoma 3611 viral oncogene homolog | |
| *BCKDHA* | branched chain keto acid dehydrogenase E1, alpha polypeptide | |
| *COQ3* | coenzyme Q3 homolog, methyltransferase (*S. cerevisiae*) | |
| *COX6A1* | cytochrome c oxidase subunit VIa polypeptide 1 | |
| *DGAT1* | diacylglycerol O-acyltransferase 1 | |
| *DNAJC15* | DnaJ (Hsp40) homolog, subfamily C, member 15 | |
| *ECHS1* | enoyl CoA hydratase, short chain, 1, mitochondrial | |
| *ETFB* | electron-transfer-flavoprotein, beta polypeptide | |
| *GPX4* | glutathione peroxidase 4 | |
| *IDH3G* | isocitrate dehydrogenase 3 (NAD^+^) gamma | |
| *MDH2* | malate dehydrogenase 2, NAD (mitochondrial) | |
| *NDUFAB1* | NADH dehydrogenase (ubiquinone) 1, alpha/beta subcomplex, 1, 8kDa | |
| *NMT1* | N-myristoyltransferase 1 | |
| *PEMT* | phosphatidylethanolamine N-methyltransferase | |
| *PEX14* | peroxisomal biogenesis factor 14 | |
| *PFKL* | phosphofructokinase, liver | |
| *PHYH* | phytanoyl-CoA 2-hydroxylase | |
| *POR* | P450 (cytochrome) oxidoreductase | |
| *PREB* | prolactin regulatory element binding | |
| *QDPR* | quinoid dihydropteridine reductase | |
| *SLC25A10* | solute carrier family 25 (mitochondrial carrier; dicarboxylate transporter), member 10 | |
| *SOD1* | superoxide dismutase 1, soluble | |
| *TALDO1* | transaldolase 1 | |
| *TST* | thiosulfate sulfurtransferase (rhodanese) | |
| *UQCR11* | ubiquinol-cytochrome c reductase, complex III subunit XI | |
| *UQCRQ* | ubiquinol-cytochrome c reductase, complex III subunit VII, 9.5kDa | |
| **List of Fatty Acid Metabolism (M5935) genes with core enrichment in 2 datasets (GSE10797 and GSE20437)** | | |
| Gene | Name | |
| *ACAA1* | acetyl-CoA acyltransferase 1 | |
| *ACADVL* | acyl-CoA dehydrogenase, very long chain | |
| *ADSL* | adenylosuccinate lyase | |
| *ALDOA* | aldolase A, fructose-bisphosphate | |
| *CA6* | carbonic anhydrase VI | |
| *CEL* | carboxyl ester lipase (bile salt-stimulated lipase) | |
| *ECHS1* | enoyl CoA hydratase, short chain, 1, mitochondrial | |
| *ECI1* | enoyl-CoA delta isomerase 1 | |
| *EPHX1* | epoxide hydrolase 1, microsomal (xenobiotic) | |
| *FH* | fumarate hydratase | |
| *GLUL* | glutamate-ammonia ligase | |
| *HMGCL* | 3-hydroxymethyl-3-methylglutaryl-CoA lyase | |
| *HSD17B10* | hydroxysteroid (17-beta) dehydrogenase 10 | |
| *IDH3B* | isocitrate dehydrogenase 3 (NAD+) beta | |
| *IDH3G* | isocitrate dehydrogenase 3 (NAD+) gamma | |
| *MDH2* | malate dehydrogenase 2, NAD (mitochondrial) | |
| *MIF* | macrophage migration inhibitory factor (glycosylation-inhibiting factor) | |
| *NTHL1* | nth endonuclease III-like 1 (E. coli) | |
| *PDHB* | pyruvate dehydrogenase (lipoamide) beta | |
| *UROD* | uroporphyrinogen decarboxylase | |
| *UROS* | uroporphyrinogen III synthase | |
| **List of DNA Repair (M5898) genes with core enrichment in 2 datasets (GSE10797 and GSE20437)** | | |
| **Gene** | **Name** | |
| *AAAS* | achalasia, adrenocortical insufficiency, alacrimia | |
| *ADRM1* | adhesion regulating molecule 1 | |
| *APRT* | adenine phosphoribosyltransferase | |
| *BCAM* | basal cell adhesion molecule (Lutheran blood group) | |
| *BRF2* | BRF2, subunit of RNA polymerase III transcription initiation factor, BRF1-like | |
| *CANT1* | calcium activated nucleotidase 1 | |
| *COX17* | COX17 cytochrome c oxidase assembly homolog (*S. cerevisiae*) | |
| *EDF1* | endothelial differentiation-related factor 1 | |
| *ERCC1* | excision repair cross-complementing rodent repair deficiency, complementation group 1 (includes overlapping antisense sequence) | |
| *ERCC2* | excision repair cross-complementing rodent repair deficiency, complementation group 2 | |
| *ERCC3* | excision repair cross-complementing rodent repair deficiency, complementation group 3 | |
| *GMPR2* | guanosine monophosphate reductase 2 | |
| *GPX4* | glutathione peroxidase 4 | |
| *GTF2F1* | general transcription factor IIF, polypeptide 1, 74kDa | |
| *GUK1* | guanylate kinase 1 | |
| *NME3* | NME/NM23 nucleoside diphosphate kinase 3 | |
| *NME4* | NME/NM23 nucleoside diphosphate kinase 4 | |
| *NPR2* | natriuretic peptide receptor B/guanylate cyclase B (atrionatriuretic peptide receptor B) | |
| *POLA2* | polymerase (DNA directed), alpha 2, accessory subunit | |
| *POLD3* | polymerase (DNA-directed), delta 3, accessory subunit | |
| *POLR1D* | polymerase (RNA) I polypeptide D, 16kDa | |
| *POLR2E* | polymerase (RNA) II (DNA directed) polypeptide E, 25kDa | |
| *POLR2I* | polymerase (RNA) II (DNA directed) polypeptide I, 14.5kDa | |
| *POLR2J* | polymerase (RNA) II (DNA directed) polypeptide J, 13.3kDa | |
| *RAE1* | RAE1 RNA export 1 homolog (S. pombe) | |
| *RALA* | v-ral simian leukemia viral oncogene homolog A (ras related) | |
| *SF3A3* | splicing factor 3a, subunit 3, 60kDa | |
| *SSRP1* | structure specific recognition protein 1 | |
| *SURF1* | surfeit 1 | |
| *TAF1C* | TATA box binding protein (TBP)-associated factor, RNA polymerase I, C, 110kDa | |
| *TAF6* | TAF6 RNA polymerase II, TATA box binding protein (TBP)-associated factor, 80kDa | |
| *TMED2* | transmembrane emp24 domain trafficking protein 2 | |
| *VPS28* | vacuolar protein sorting 28 homolog (*S. cerevisiae*) | |
| *VPS37B* | vacuolar protein sorting 37 homolog B (*S. cerevisiae*) | |
