# Supplementary material for: TRPM4 is overexpressed in breast cancer associated with estrogen response and epithelial-mesenchymal transition gene sets
Source: PLoS One. 2020 Jun 2;15(6):e0233884. doi: 10.1371/journal.pone.0233884 (PMC7266295; doi:10.1371/journal.pone.0233884)
Supplement: S4 Table — (DOCX) [file pone.0233884.s006.docx]

**S4 Table. List of consensus genes contributing to the enrichment of estrogen responses (Hallmark ID: M5906 and M5907) and EMT gene sets (M5930) according to *TRPM4* expression in breast cancer datasets (GEO ID: GSE54002, GSE20685 and GSE23720).**

| **List of Estrogen Response Early (ID: M5907) genes with core enrichment in all 3 datasets (GSE54002, GSE20685 and GSE23720)** | |
| --- | --- |
| **Gene** | **Name** |
| *ABAT* | 4-aminobutyrate aminotransferase |
| *ADCY9* | adenylate cyclase 9 |
| *ANXA9* | annexin A9 |
| *AR* | androgen receptor |
| *BLVRB* | biliverdin reductase B (flavin reductase (NADPH)) |
| *CA12* | carbonic anhydrase XII |
| *CANT1* | calcium activated nucleotidase 1 |
| *CISH* | cytokine inducible SH2-containing protein |
| *CLDN7* | claudin 7 |
| *ELF1* | E74-like factor 1 (ets domain transcription factor) |
| *ELOVL5* | ELOVL fatty acid elongase 5 |
| *ESRP2* | epithelial splicing regulatory protein 2 |
| *FASN* | fatty acid synthase |
| *GFRA1* | GDNF family receptor alpha 1 |
| *GREB1* | growth regulation by estrogen in breast cancer 1 |
| *HES1* | hairy and enhancer of split 1, (Drosophila) |
| *IGF1R* | insulin-like growth factor 1 receptor |
| *IGFBP4* | insulin-like growth factor binding protein 4 |
| *INHBB* | inhibin, beta B |
| *KDM4B* | Lysine (K)-specific demethylase 4B |
| *KRT18* | keratin 18 |
| *KRT19* | keratin 19 |
| *KRT8* | keratin 8 |
| *MAPT* | microtubule-associated protein tau |
| *MAST4* | microtubule associated serine/threonine kinase family member 4 |
| *MLPH* | melanophilin |
| *MUC1* | mucin 1, cell surface associated |
| *MYB* | v-myb myeloblastosis viral oncogene homolog (avian) |
| *MYOF* | myoferlin |
| *OLFML3* | olfactomedin-like 3 |
| *P2RY2* | purinergic receptor P2Y, G-protein coupled, 2 |
| *PDZK1* | PDZ domain containing 1 |
| *PEX11A* | peroxisomal biogenesis factor 11 alpha |
| *PGR* | progesterone receptor |
| *RAB17* | RAB17, member RAS oncogene family |
| *RAB31* | RAB31, member RAS oncogene family |
| *RARA* | retinoic acid receptor, alpha |
| *REEP1* | receptor accessory protein 1 |
| *SCNN1A* | sodium channel, non-voltage-gated 1 alpha subunit |
| *SEC14L2* | SEC14-like 2 (*S. cerevisiae*) |
| *SLC1A1* | solute carrier family 1 (neuronal/epithelial high affinity glutamate transporter,  system Xag), member 1 |
| *SLC1A4* | solute carrier family 1 (glutamate/neutral amino acid transporter), member 4 |
| *SLC22A5* | solute carrier family 22 (organic cation/carnitine transporter), member 5 |
| *SLC37A1* | solute carrier family 37 (glycerol-3-phosphate transporter), member 1 |
| *SLC9A3R1* | solute carrier family 9, subfamily A (NHE3, cation proton antiporter 3), member  3 regulator 1 |
| *STC2* | stanniocalcin 2 |
| *SYBU* | syntabulin (syntaxin-interacting) |
| *TBC1D30* | TBC1 domain family, member 30 |
| *TFF1* | trefoil factor 1 |
| *TFF3* | trefoil factor 3 (intestinal) |
| *THSD4* | thrombospondin, type I, domain containing 4 |
| *TJP3* | tight junction protein 3 (zona occludens 3) |
| *TPBG* | trophoblast glycoprotein |
| *TTC39A* | tetratricopeptide repeat domain 39A |
| *WFS1* | Wolfram syndrome 1 (wolframin) |
| *WWC1* | WW and C2 domain containing 1 |
| *XBP1* | X-box binding protein 1 |
| **List of Estrogen Response Late (ID: M5907) genes with core enrichment in all 3 datasets (GSE54002, GSE20685 and GSE23720)** | |
| **Gene** | **Name** |
| *AGR2* | anterior gradient 2 homolog (Xenopus laevis) |
| *ANXA9* | annexin A9 |
| *BLVRB* | biliverdin reductase B (flavin reductase (NADPH)) |
| *CA12* | carbonic anhydrase XII |
| *CACNA2D2* | calcium channel, voltage-dependent, alpha 2/delta subunit 2 |
| *CISH* | cytokine inducible SH2-containing protein |
| *CXCL14* | chemokine (C-X-C motif) ligand 14 |
| *CYP4F11* | cytochrome P450, family 4, subfamily F, polypeptide 11 |
| *DCXR* | dicarbonyl/L-xylulose reductase |
| *DNAJC1* | DnaJ (Hsp40) homolog, subfamily C, member 1 |
| *DNAJC12* | DnaJ (Hsp40) homolog, subfamily C, member 12 |
| *ELOVL5* | ELOVL fatty acid elongase 5 |
| *EMP2* | epithelial membrane protein 2 |
| *FGFR3* | fibroblast growth factor receptor 3 |
| *GALE* | UDP-galactose-4-epimerase |
| *IGFBP4* | insulin-like growth factor binding protein 4 |
| *KRT19* | keratin 19 |
| *LLGL2* | lethal giant larvae homolog 2 (Drosophila) |
| *MAPT* | microtubule-associated protein tau |
| *MYB* | v-myb myeloblastosis viral oncogene homolog (avian) |
| *MYOF* | myoferlin |
| *PDZK1* | PDZ domain containing 1 |
| *PGR* | progesterone receptor |
| *PLXNB1* | plexin B1 |
| *PRLR* | prolactin receptor |
| *PTGER3* | prostaglandin E receptor 3 (subtype EP3) |
| *RAB31* | RAB31, member RAS oncogene family |
| *RABEP1* | rabaptin, RAB GTPase binding effector protein 1 |
| *SCNN1A* | sodium channel, non-voltage-gated 1 alpha subunit |
| *SCUBE2* | signal peptide, CUB domain, EGF-like 2 |
| *SLC1A4* | solute carrier family 1 (glutamate/neutral amino acid transporter), member 4 |
| *SLC22A5* | solute carrier family 22 (organic cation/carnitine transporter), member 5 |
| *SLC9A3R1* | solute carrier family 9, subfamily A (NHE3, cation proton antiporter 3),  member 3 regulator 1 |
| *ST6GALNAC2* | ST6 (alpha-N-acetyl-neuraminyl-2,3-beta-galactosyl-1,3)-N- acetylgalactosaminide alpha-2,6-sialyltransferase 2 |
| *SULT2B1* | sulfotransferase family, cytosolic, 2B, member 1 |
| *TFF1* | trefoil factor 1 |
| *TFF3* | trefoil factor 3 (intestinal) |
| *TJP3* | tight junction protein 3 (zona occludens 3) |
| *TPBG* | trophoblast glycoprotein |
| *TPSAB1* | tryptase alpha/beta 1 |
| *TSTA3* | tissue specific transplantation antigen P35B |
| *WFS1* | Wolfram syndrome 1 (wolframin) |
| *XBP1* | X-box binding protein 1 |
| **List of Epithelial-Mesenchymal Transition (ID: M5930) genes with core enrichment in all 3 datasets (GSE54002, GSE20685 and GSE23720)** | |
| **Gene** | **Name** |
| *CAP2* | CAP, adenylate cyclase-associated protein, 2 (yeast) |
| *COL11A1* | collagen, type XI, alpha 1 |
| *COL16A1* | collagen, type XVI, alpha 1 |
| *COL1A1* | collagen, type I, alpha 1 |
| *COL1A2* | collagen, type I, alpha 2 |
| *COL5A1* | collagen, type V, alpha 1 |
| *COL6A3* | collagen, type VI, alpha 3 |
| *COL8A2* | collagen, type VIII, alpha 2 |
| *COMP* | cartilage oligomeric matrix protein |
| *ECM1* | extracellular matrix protein 1 |
| *EFEMP2* | EGF containing fibulin-like extracellular matrix protein 2 |
| *FAP* | fibroblast activation protein, alpha |
| *FSTL3* | follistatin-like 3 (secreted glycoprotein) |
| *GPC1* | glypican 1 |
| *HTRA1* | HtrA serine peptidase 1 |
| *IGFBP2* | insulin-like growth factor binding protein 2, 36kDa |
| *IGFBP4* | insulin-like growth factor binding protein 4 |
| *ITGB5* | integrin, beta 5 |
| *LOXL1* | lysyl oxidase-like 1 |
| *LRRC15* | leucine rich repeat containing 15 |
| *MATN3* | matrilin 3 |
| *MXRA5* | matrix-remodelling associated 5 |
| *MYL9* | myosin, light chain 9, regulatory |
| *PCOLCE* | procollagen C-endopeptidase enhancer |
| *PDGFRB* | platelet-derived growth factor receptor, beta polypeptide |
| *PMEPA1* | prostate transmembrane protein, androgen induced 1 |
| *RHOB* | ras homolog family member B |
| *SDC4* | syndecan 4 |
| *SPOCK1* | sparc/osteonectin, cwcv and kazal-like domains proteoglycan (testican) 1 |
| *THY1* | Thy-1 cell surface antigen |
| *TIMP3* | TIMP metallopeptidase inhibitor 3 |
