# Supplementary material for: TRPM4 is overexpressed in breast cancer associated with estrogen response and epithelial-mesenchymal transition gene sets
Source: PLoS One. 2020 Jun 2;15(6):e0233884. doi: 10.1371/journal.pone.0233884 (PMC7266295; doi:10.1371/journal.pone.0233884)
Supplement: S5 Table — (DOCX) [file pone.0233884.s007.docx]

**S5 Table. List of consensus genes contributing to the enrichment of Myogenesis gene set (Hallmark ID: M5909) according to *TRPM4* expression in breast cancer datasets (GEO ID: GSE20685 and GSE23720).**

| **List of Myogenesis (ID: M5909) genes with core enrichment in 2 datasets**  **(GSE20685 and GSE23720)** | |
| --- | --- |
| *SPDEF* | SAM pointed domain containing ets transcription factor |
| *ITGB5* | integrin, beta 5 |
| *COL1A1* | collagen, type I, alpha 1 |
| *CRAT* | carnitine O-acetyltransferase |
| *TSC2* | tuberous sclerosis 2 |
| *AGRN* | agrin |
| *AEBP1* | AE binding protein 1 |
| *DMPK* | Dystrophia myotonica-protein kinase |
| *SH2B1* | SH2B adaptor protein 1 |
| *COL3A1* | collagen, type III, alpha 1 |
| *ERBB3* | v-erb-b2 erythroblastic leukemia viral oncogene homolog 3 (avian) |
| *REEP1* | receptor accessory protein 1 |
| *MYO1C* | myosin IC |
| *COL6A2* | collagen, type VI, alpha 2 |
| *COL6A3* | collagen, type VI, alpha 3 |
| *PPP1R3C* | protein phosphatase 1, regulatory subunit 3C |
| *SGCD* | sarcoglycan, delta (35kDa dystrophin-associated glycoprotein) |
| *VIPR1* | vasoactive intestinal peptide receptor 1 |
| *ADAM12* | ADAM metallopeptidase domain 12 |
| *PLXNB2* | plexin B2 |
| *NQO1* | NAD(P)H dehydrogenase, quinone 1 |
| *BHLHE40* | basic helix-loop-helix family, member e40 |
| *AKT2* | V-akt murine thymoma viral oncogene homolog 2 |
| *OCEL1* | occludin/ELL domain containing 1 |
| *ADCY9* | adenylate cyclase 9 |
| *SORBS3* | sorbin and SH3 domain containing 3 |
| *AGL* | amylo-alpha-1, 6-glucosidase, 4-alpha-glucanotransferase |
| *PDLIM7* | PDZ and LIM domain 7 (enigma) |
| *ATP6AP1* | ATPase, H^+^ transporting, lysosomal accessory protein 1 |
| *SYNGR2* | synaptogyrin 2 |
| *MAPRE3* | microtubule-associated protein, RP/EB family, member 3 |
| *COX7A1* | cytochrome c oxidase subunit VIIa polypeptide 1 (muscle) |
| *CASQ1* | calsequestrin 1 (fast-twitch, skeletal muscle) |
| *TGFB1* | transforming growth factor, beta 1 |
| *MEF2D* | myocyte enhancer factor 2D |
| *TCAP* | titin-cap (telethonin) |
| *STC2* | stanniocalcin 2 |
| *GADD45B* | growth arrest and DNA-damage-inducible, beta |
| *RIT1* | Ras-like without CAAX 1 |
| *PVALB* | parvalbumin |
| *CACNG1* | calcium channel, voltage-dependent, gamma subunit 1 |
| *HRC* | histidine rich calcium binding protein |
| *CKB* | creatine kinase, brain |
| *ENO3* | enolase 3 (beta, muscle) |
| *SMTN* | smoothelin |
| *SGCA* | sarcoglycan, alpha (50kDa dystrophin-associated glycoprotein) |
| *DES* | desmin |
| *BDKRB2* | bradykinin receptor B2 |
| *FST* | follistatin |
| *TAGLN* | transgelin |
| *CSRP3* | cysteine and glycine-rich protein 3 (cardiac LIM protein) |
| *PGAM2* | phosphoglycerate mutase 2 (muscle) |
| *TNNT1* | troponin T type 1 (skeletal, slow) |
| *FLII* | flightless I homolog (Drosophila) |
| *LAMA2* | laminin, alpha 2 |
| *ITGB1* | integrin, beta 1 (fibronectin receptor, beta polypeptide, antigen CD29 includes MDF2, MSK12) |
| *ACTA1* | actin, alpha 1, skeletal muscle |
| *FHL1* | four and a half LIM domains 1 |
| *GAA* | glucosidase, alpha; acid |
| *ABLIM1* | actin binding LIM protein 1 |
| *IGFBP7* | insulin-like growth factor binding protein 7 |
| *CASQ2* | calsequestrin 2 (cardiac muscle) |
| *SPTAN1* | spectrin, alpha, non-erythrocytic 1 |
