# Supplementary material for: TRPM4 is overexpressed in breast cancer associated with estrogen response and epithelial-mesenchymal transition gene sets
Source: PLoS One. 2020 Jun 2;15(6):e0233884. doi: 10.1371/journal.pone.0233884 (PMC7266295; doi:10.1371/journal.pone.0233884)
Supplement: S6 Table — (DOCX) [file pone.0233884.s008.docx]

**S6 Table. IHC of TRPM4 or AR in breast cancer cases according to Human Protein Atlas database.**

| **TRPM4 IHC (HPA041169 antibody)** | | | | | | **AR IHC (CAB065764 antibody)** | | | | | |
| --- | --- | --- | --- | --- | --- | --- | --- | --- | --- | --- | --- |
| **No** | **Patient ID** | **Gender & age** | **Frequency** | **Intensity** | **Cellular location** | **No** | **Patient ID** | **Gender & age** | **Frequency** | **Intensity** | **Cellular location** |
| 1 | 1874 | F, 80 | >75% | Moderate | Cytoplasmic/  membranous | 1 | 1874 | F, 80 | >75% | Moderate | Nuclear |
| 2 | 1910 | F, 61 | >75% | Moderate | Cytoplasmic/  membranous | 2 | 1910 | F, 61 | 25-75% | Weak | Nuclear |
| 3 | 2805 | F, 59 | <25% | Weak | Cytoplasmic/  membranous | 3 | 2805 | F, 59 | >75% | Moderate | Nuclear |
| 4 | 2160 | F, 83 | - | Negative | - | 4 | 2160 | F, 83 | <25% | Moderate | Nuclear |
| 5 | 1775 | F, 55 | 25-75% | Moderate | Cytoplasmic/  membranous | 5 | 4852 | F, 85 | >75% | Strong | Nuclear |
| 6 | 1939 | F, 87 | >75% | Moderate | Cytoplasmic/  membranous | 6 | 4229 | F, 66 | >75% | Strong | Nuclear |
| 7 | 1785 | F, 93 | >75% | Moderate | Cytoplasmic/  membranous | 7 | 4653 | F, 84 | 25-75% | Strong | Nuclear |
| 8 | 2565 | F, 51 | >75% | Moderate | Cytoplasmic/  membranous | 8 | 4789 | F, 49 | >75% | Moderate | Nuclear |
| 9 | 2091 | F, 40 | 25-75% | Weak | Cytoplasmic/  membranous | 9 | 643 | F, 62 | >75% | Moderate | Nuclear |
| 10 | 2428 | F, 75 | 25-75% | Weak | Cytoplasmic/  membranous | 10 | 4193 | F, 43 | >75% | Moderate | Nuclear |
| 11 | 3546 | F, 58 | <25% | Weak | Cytoplasmic/  membranous | 11 | 1268 | F, 54 | >75% | Moderate | Nuclear |
| - | - | - | - | - | - | 12 | 2392 | F, 27 | <25% | Weak | Nuclear |

Cases with the same Patient ID are gray-highlighted.
